# Regiochemistry of Donor Dendrons Controls the Performance of Thermally Activated Delayed Fluorescence Dendrimer Emitters for High Efficiency Solution‐Processed Organic Light‐Emitting Diodes

**DOI:** 10.1002/advs.202201470

**Published:** 2022-04-25

**Authors:** Dianming Sun, Rishabh Saxena, Xiaochun Fan, Stavros Athanasopoulos, Eimantas Duda, Ming Zhang, Sergey Bagnich, Xiaohong Zhang, Eli Zysman‐Colman, Anna Köhler

**Affiliations:** ^1^ Institute of Functional Nano & Soft Materials (FUNSOM) and Jiangsu Key Laboratory for Carbon‐Based Functional Materials & Devices Joint International Research Laboratory of Carbon‐Based Functional Materials and Devices Soochow University Suzhou Jiangsu 215123 P. R. China; ^2^ Organic Semiconductor Centre EaStCHEM School of Chemistry University of St Andrews St Andrews KY16 9ST UK; ^3^ Soft Matter Optoelectronics Soft Matter Optoelectronics and Bavarian Polymer Institute (BPI) University of Bayreuth Universitätsstraße 30 95447 Bayreuth Germany; ^4^ Departamento de Física Universidad Carlos III de Madrid Avenida Universidad 30, 28911 Leganés Madrid Spain; ^5^ Bayreuth Institute of Macromolecular Research (BIMF) University of Bayreuth Universitätstraße 30 95447 Bayreuth Germany

**Keywords:** carbazole, external quantum efficiency, OLEDs, solution‐processing, TADF dendrimers, triazine

## Abstract

The potential of dendrimers exhibiting thermally activated delayed fluorescence (TADF) as emitters in solution‐processed organic light‐emitting diodes (OLEDs) has to date not yet been realized. This in part is due to a poor understanding of the structure–property relationship in dendrimers where reports of detailed photophysical characterization and mechanism studies are lacking. In this report, using absorption and solvatochromic photoluminescence studies in solution, the origin and character of the lowest excited electronic states in dendrimers with multiple dendritic electron‐donating moieties connected to a central electron‐withdrawing core via a *para*‐ or a *meta*‐phenylene bridge is probed. Characterization of host‐free OLEDs reveals the superiority of *meta*‐linked dendrimers as compared to the already reported *para*‐analogue. Comparative temperature‐dependent time‐resolved solid‐state photoluminescence measurements and quantum chemical studies explore the effect of the substitution mode on the TADF properties and the reverse intersystem crossing (RISC) mechanism, respectively. For TADF dendrimers with similarly small ∆*E*_ST_, it is observed that RISC can be enhanced by the regiochemistry of the donor dendrons due to control of the reorganization energies, which is a heretofore unexploited strategy that is distinct from the involvement of intermediate triplet states through a nonadiabatic (vibronic) coupling with the lowest singlet charge transfer state.

## Graphical abstract

A series of thermally activated delayed fluorescence (TADF) dendrimers is developed. The study reveals that the reorganization energies and thus the reverse intersystem crossing rate can be controlled by the regiochemistry of the donor dendrons. A two‐fold improved external quantum efficiency in solution‐processed organic light‐emitting diodes is demonstrated using *meta*‐connected versus a *para*‐connected TADF dendrimers as emitters.

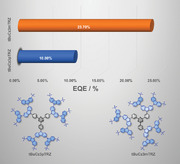

## 1 Introduction

Since 2012, organic compounds showing thermally activated delayed fluorescence (TADF) have generated intense interest as replacement emitter materials for noble‐metal based phosphorescent complexes in organic light emitting diodes (OLEDs)^[^ ^1^ ^]^ as TADF‐based materials are sustainable and less expensive to synthesize while also allowing for 100% internal quantum efficiency (IQE) by recruiting both singlet and triplet excitons for light emission. Since the first organic TADF OLED was reported in 2009,^[^ ^2^ ^]^ the design of purely organic TADF‐based emitters has evolved rapidly, and vacuum‐deposited OLEDs have demonstrated impressive maximum external quantum efficiencies (EQE_max_) exceeding 20% for each of the three primary colors. Further, low molecular weight small molecule TADF OLEDs have satisfied critical industry requirements in terms of color, efficiency, and device lifetime, and so can be envisioned to replace phosphorescent emitters in commercial display panels.

Though generally OLEDs are fabricated by vacuum deposition, the cost of fabrication coupled with the inefficient use of materials and limitations on the size of pixel are all detracting features of this technology. An alternative fabrication process that is cost‐ and materials‐efficient is solution‐processing. Solution‐processing OLED fabrication techniques such as spin‐coating^[^ ^3^ ^]^ and ink‐jet printing^[^ ^4^ ^]^ are advantageous as the device architecture is simpler, less material is lost during film formation and large‐area display fabrication becomes much more feasible. What is required are devices that exhibit comparable performance metrics as vacuum‐deposited devices, and this is underpinned by high‐performance solution‐processable emitter materials. Unlike small molecules, dendrimers and polymers allow for easy solution‐processed manufacture of large‐area devices because of their superior film‐forming ability, excellent thermal and morphological stability, and high affinity for substrates.^[^ ^5^ ^]^ The molecular weight distribution of polymers, however, normally leads to batch‐to‐batch deviation of their (photo)physical properties. Unlike polymers, dendrimers have a defined molecular weight. Moreover, in general, most TADF dendrimers do not need to be dispersed into host matrices to suppress concentration or aggregation‐caused quenching of the emission and/or exciton annihilation. Thus, nondoped OLEDs can be fabricated resulting in a simplified device architecture.

To date, there have been only a handful of reports using TADF dendrimers,^[^ ^6^ ^]^ most of which disclose devices that possess efficiencies that are far from the state‐of‐the‐art (Table S1, Supporting Information) of small molecule TADF OLEDs. Slow progress due to a paucity of examples of TADF dendrimers and a poor understanding of their photophysics have hampered the development of dendrimer TADF OLEDs. Most of the reported TADF dendrimers adopt multiple dendritic electron‐donating moieties that are connected to a central electron‐withdrawing core structure through either conjugated or nonconjugated linkers. Delocalization of the distribution of the electron density of the highest occupied molecular orbital (HOMO) over the donor dendrons, combined with confinement of the electron density in the lowest unoccupied molecular orbital (LUMO) within the acceptor core results in a small exchange integral and an efficient reverse intersystem crossing (RISC) rate. Furthermore, the presence of many donors results in a closer packing of dendrons around acceptor core that helps to improve the charge injection ability of the material and reduce the probability of triplet‐triplet annihilation (TTA) in the neat film.^[^ ^7^ ^]^ As well, the presence of a large number of donor dendrons around the central acceptor can effectively increase the transition integral for large radiative decay rate.^[^ ^8^ ^]^

**Figure 1:**
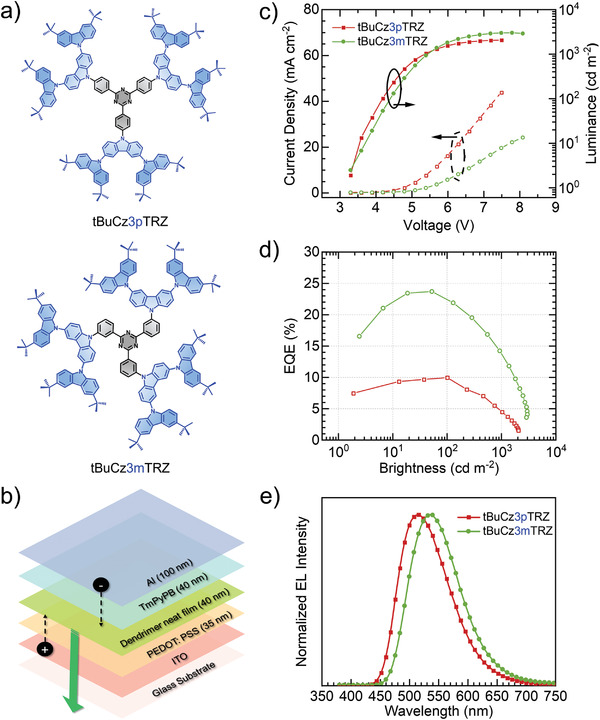
a) Molecular structures of **tBuCz3pTRZ** and **tBuCz3mTRZ**. b) Device configuration of the nondoped solution processed OLEDs. c) Current density–voltage–luminance plot, d) EQE versus brightness curve, and e) electroluminescence (EL) spectra for the nondoped OLEDs based on **tBuCz3pTRZ** and **tBuCz3mTRZ**.

The first TADF dendrimers devised were based on trisubstituted dendronized carbazole donors distributed about a central triphenyltriazine (**TRZ**) unit.^[^ ^6a^ ^]^ Yamamoto et al.^[^ ^6e^ ^]^ reported that the performance of the “host‐free” devices could be improved by decorating each of the peripheral carbazoles with *tert*‐butyl groups (**tBuG2TAZ**). The chemical structure of one of this second generation carbazole‐triazine dendrimer, **tBuCz3pTRZ**, is shown in **Figure** **1**a. It consists of a **TRZ** acceptor linked to the 3,3″,6,6″‐tetrakis(*tert*‐butyl‐9′H‐9,3′:6′,9″‐tercarbazole) (**2GtBuCz**) donors via a *para‐*phenylene bridge. **tBuCz3pTRZ** shows 100% photoluminescence quantum yield (PLQY) in toluene solution but suffers significant quenching in the solid state, especially in nondoped films. Most TADF dendrimers adopt donor‐*para*‐*π*‐acceptor architectures where the donor and acceptor units are *para*‐connected about a phenylene bridging moiety. Despite the small Δ*E*_ST_ values that emanate from this design, the performance of the OLEDs is not comparable to those based on small‐molecule TADF OLEDs. Consequently, there is a disconnect between this key parameter and the performance of the device and thus, it is important to understand more fundamentally the photophysics of TADF dendrimers and their relationship to both the structure and the device performance.

In general, the relative position of donors and acceptor has a significant effect on the TADF properties.^[^ ^9^ ^]^ Different substitution modes influence the spatial overlap of the HOMO hole density and the lowest unoccupied molecular orbital (LUMO) electron density distributions. This leads to different Δ*E*_ST_ values, thus affecting the efficiency and nature of the RISC process of the TADF materials. This motivated us to compare the *para*‐bridged dendrimer with its *meta*‐based analogues to assess the influence of the *para‐* versus *meta‐*linked dendrons. This information is important because the correlation of the photophysical and device properties with the molecular structure is crucial for the design of highly efficient TADF dendrimers. In the present work, we synthesized a series of TADF dendrimers with a *meta‐*phenylene bridge and investigated their photophysics in detail to build a fundamental understanding of the origin and nature of the lowest energy excited states.

## 2 Results

### 2.1 Device Characterization for *para‐* versus *meta‐*Linked Dendrimers

To assess the effect of substitution position of the donor dendrons on the dendrimer emitter on the OLED metrics, firstly we synthesized the previously reported **tBuCz3pTRZ** and its *meta*‐analog **tBuCz3mTRZ** (Figure 1a) (see the Supporting Information for synthesis). The electrochemical properties were determined to evaluate the HOMO and LUMO levels. Both dendrimers possess similar electrochemical properties that translate into similar energy HOMOs of −5.41 and −5.40 eV while the LUMO levels are −2.84 and −2.82 eV for **tBuCz3pTRZ** and **tBuCz3mTRZ**, respectively (Table S2, Supporting Information). The high‐lying HOMO level should facilitate the injection of holes from the PEDOT:PSS hole‐injection layer. Simple nondoped (i.e., host‐free) devices were fabricated with a device configuration of ITO/PEDOT:PSS (35 nm)/dendrimer (40 nm)/TmPyPB (40 nm)/LiF (1 nm)/Al (100 nm). A schematic diagram illustrating the device structure is also presented in Figure 1b. Figure 1c shows the current density–voltage–luminance (*J*–*V*–*L*) curves for the devices employing **tBuCz3pTRZ** and **tBuCz3mTRZ** within the emissive layer. Devices based on these dendrimers have the same turn‐on voltage of around 3.3 V. The EQE versus brightness curves for the two devices are shown in Figure 1d. The **tBuCz3mTRZ**‐based device exhibited a maximum external quantum efficiency (EQE_max_) of 23.8% (EQE at 100 cd m^−2^, EQE_100_ = 22.2%), while the tBuCz3pTRZ‐based device showed a much more moderate EQE_max_ of 10% (which was obtained at 100 cd m^−2^), which is consistent with the reported value of 9.5%, albeit the device was fabricated with a different architecture.^[^ ^6e^ ^]^ This two‐fold improvement in the EQE_max_ increases to a factor of 3 at higher luminance where an EQE at 1000 cd m^−2^ of 15% was obtained for the **tBuCz3mTRZ**‐based device compared to 5% for **tBuCz3pTRZ**‐based device. The EL spectra of these two devices, shown in Figure 1e, have similar spectral shape with the peak, *λ*_EL_, at 516 nm for the **tBuCz3pTRZ**‐based device and 536 nm for the **tBuCz3mTRZ**‐based device.

**Figure 2:**
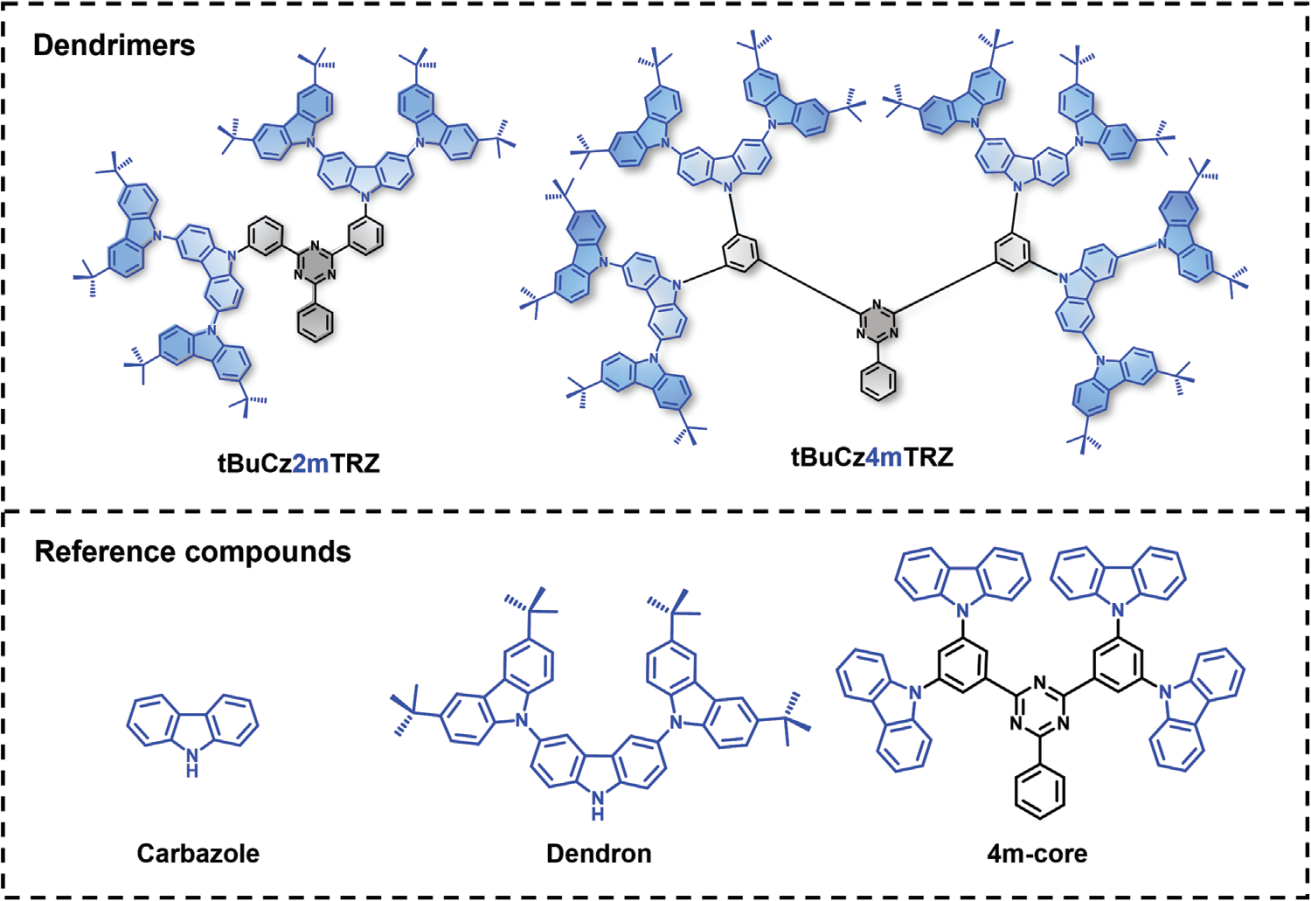
The molecular structures of the *meta*‐connected dendrimers, **tBuCz2mTRZ** and **tBuCz4mTRZ** as well as the three reference compounds, carbazole, dendron, and **4m‐core** (**tBuCz4mTRZ** without the outer tBuCz units).

To understand whether this enhanced performance can really be associated with the change in regiochemistry of the donor dendrons linked to the acceptor core, two more *meta*‐bridged dendrimers, **tBuCz2mTRZ** and **tBuCz4mTRZ**, were prepared by connecting carbazole dendron units to the acceptor TRZ core. **tBuCz2mTRZ** and **tBuCz4mTRZ** (**Figure** **2**) contain two and four dendrons, respectively, within their chemical structure and possess different symmetry compared to **tBuCz3mTRZ**. The devices were prepared using the same architecture and the OLED performance is shown in Figure S3 (Supporting Information). Intriguingly, the **tBuCz2mTRZ**‐based device (EQE_max_ = 19.9% at 23 cd m^–2^; EQE_100_ = 12.0%) as well as the **tBuCz4mTRZ**‐based device (EQE_max_ = 23.8% at 100 cd m^−2^) are also found to have higher EQEs than that of the OLED with **tBuCz3pTRZ **(Figure S3b, Supporting Information). The EQE_max_ reported here (Table S3, Supporting Information) for the *meta*‐linked dendrimers are amongst the highest values reported for solution‐processed TADF OLEDs (see Table S1 in the Supporting Information). The results establish that, for the same device architecture, these *meta*‐dendrimer‐based OLEDs show significantly better EL performance than the *para*‐dendrimer analogue, which in turn, confirms the importance of the substitution mode between the donor dendrons and the acceptor moiety in the molecular design of TADF dendrimers. However, a detailed photophysical and quantum chemical analysis is needed to understand the correlation between the OLED performance with the dendrimer structure, which is the object of this study. A separate publication is dedicated to the exploitation of this insight in the design of an optimized chemical structure that involves both, *meta*‐connected and *para*‐connected donor dendrons leading to OLEDs with EQE_max_ of 28.7%.^[^ ^10^ ^]^

### 2.2 Photophysics of the Singlet Excited States

**Figure 3:**
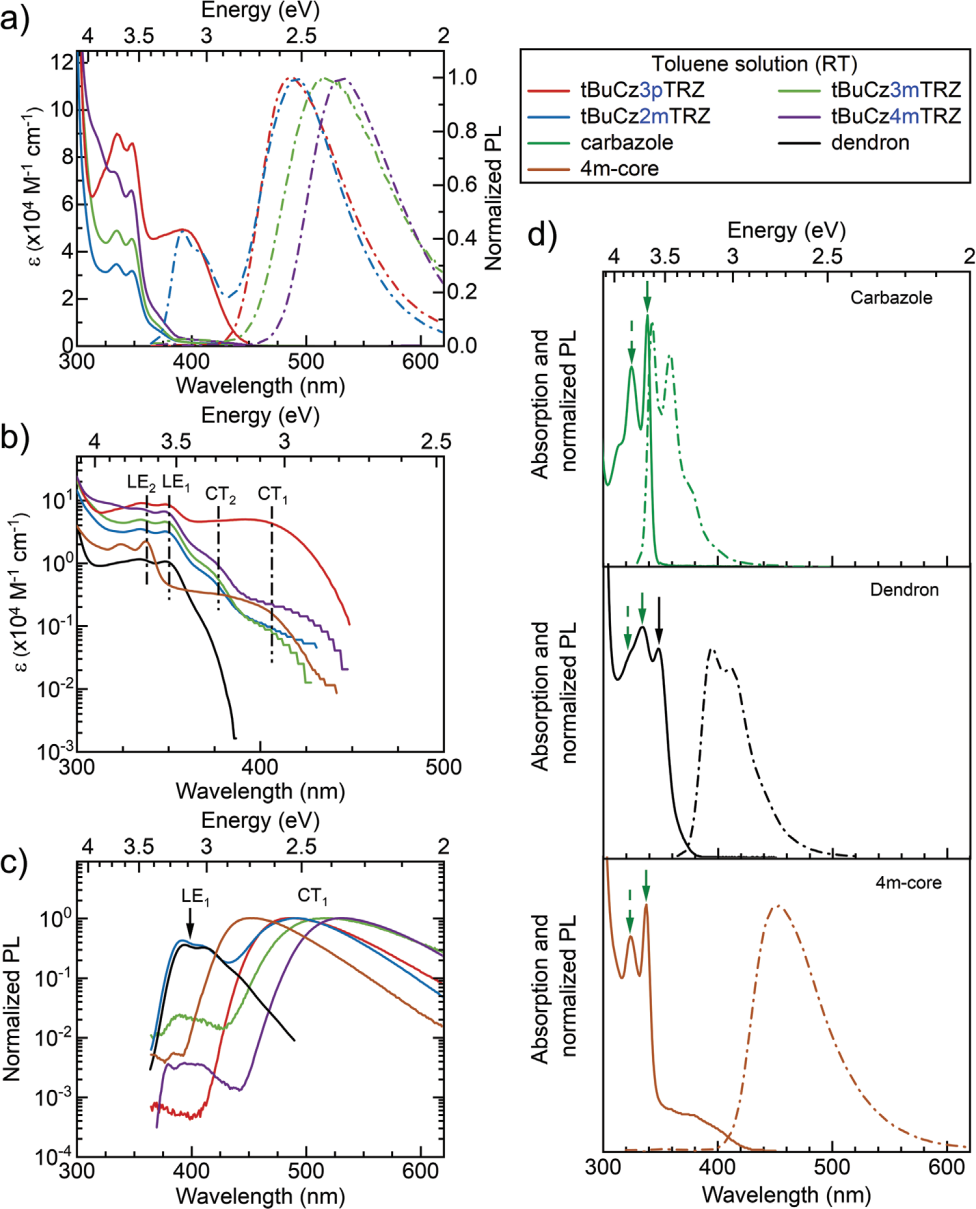
RT solution photophysics of the dendrimer and model compounds (*λ*_exc_ = 300 nm): a) Molar absorptivity and normalized emission spectra in toluene on a linear scale. b) Molar absorptivity and c) emission spectra on a log scale along with the molar absorptivity/emission spectra. d) Absorption and emission spectra (on a linear scale) of the carbazole, dendron and **4m‐core**. LE_1_, LE_2_, CT_1_, CT_2_ refer to the lower and higher energy singlet LE and CT states as described in the text. Arrows in d) refer to the 0‐0 transition (solid green) and 0–1 transition (dotted green) of the LE_2_ and the LE_1_ absorption (solid black).

Room temperature (RT) absorption and emission spectra of the dendrimers in toluene solution at low concentration are shown in **Figure** **3**a (for a linear plot) and in Figures 3b,c (for semilogarithmic plots). For reference, the absorption and emission spectra of reference compounds (see Figure 2 for chemical structures), **carbazole**, **dendron**, and **4m‐core**, are shown in Figure 3d. Two distinct bands characterize the absorption of each dendrimer. The broad band below 3.5 eV with weak‐to‐moderate intensity (Figures 3a,b) is assigned to a CT transition, while the strongly absorbing band with structured features above 3.5 eV arises from locally excited (LE) transitions on the carbazole units.

The nature of the LE band becomes evident when comparing it to the absorption spectra of the reference compounds (Figure 3d). The absorption spectra of **carbazole** shows vibrational features with peaks at 3.83 eV (324 nm) and 3.66 eV (339 nm) and the Stokes shift between the 0–0 peaks of absorption and emission is small.^[^ ^11^ ^]^ The structured features of the absorption spectrum of **4m‐core** match well in energy and spectral shape with the **carbazole** *π*–π* transition. In contrast to **carbazole** (Figure 3d, top panel), **4m‐core** exhibits a broad and weak absorption band below 3.5 eV that we attribute to absorption from a CT state between the carbazoles and the central triazine acceptor (Figure 3d, bottom panel). The emission of **4m‐core** arises from this CT state as the emission is symmetric to the CT absorption. In comparison with **carbazole** absorption, the dendron absorption is slightly red shifted with two structured peaks at 3.70 eV (335 nm) and 3.55 eV (350 nm), and a weak shoulder at 3.84 (323 nm). In addition, the relative intensity of the vibrational peak at 3.70 eV (335 nm) with respect to the 0–0 vibration peak at 3.55 eV is different from that of the **carbazole** absorption. Thus, we can conclude that the dendron absorption is comprised of two vertical excitation processes corresponding to: i) a lower energy absorption from an excitation that is localized on the central carbazole (denoted as LE_1_ and indicated here by the black arrow); and ii) a higher energy absorption from the excitation localized on the outer, individual carbazoles (referred to as LE_2_ and indicated here by the green arrows). Due to the prevailing substitution pattern of the donor dendron we expect the central carbazole to be more electron‐rich than the peripheral carbazoles, so that its excited state is slightly lower in energy. From the absorption spectra, each of the dendrimers, **tBuCz3pTRZ**, **tBuCz3mTRZ**, **tBuCz2mTRZ**, and **tBuCz4mTRZ**, exhibits similar structured features that resemble those of the dendron. Therefore, it is affirmed that their absorption spectra are dominated by the same LE_1_ and LE_2_ states as from the dendron. For clarity, they are indicated in Figure 3b. Furthermore, as shown in Figure 3b, the molar absorptivity of the bands in the range 3.5–3.8 eV increases from **tBuCz2mTRZ** to **tBuCz3mTRZ** to **tBuCz4mTRZ**. This is consistent with the presence of an increasing number of donor dendron units in their chemical structure. Similar behavior in the absorption spectra have been reported in the literature;^[^ ^6a,b,e^ ^]^ however, the spectral features around 300–400 nm have either not been discussed or have been ascribed to intramolecular CT absorption.^[^ ^6b^ ^]^

In addition to the structured dendron emission, **tBuCz3pTRZ** is characterized by the presence of a strong, broad absorption band in the range of 2.8–3.4 eV, which we ascribe to a CT transition (and refer to as CT_1_) from the dendron donors to the triazine (TRZ) acceptor. However, when the *para*‐connected donor dendron is replaced by the *meta*‐connected donor dendron this broad feature remains approximately at the same energy (centered roughly at 3.05 eV), but the extinction coefficient is reduced by over an order of magnitude. The *meta*‐connection compounds are also characterized by a higher energy broad, unstructured absorption band centered at 3.3 eV (labelled CT_2_) with a larger molar extinction coefficient than the lower energy CT_1_ band. The comparable extinction coefficient of CT_1_ band and the LE absorption features in **tBuCz3pTRZ** indicates a significant overlap of electron and hole wavefunctions, and consequently some admixture of transitions with both *π*–π* and CT_1_ character. By comparison with the absorption features of the *meta‐*connection dendrimers, it is evident that a CT_2_ state is present in **tBuCz3pTRZ** as well; however, the strong extinction coefficient of the CT_1_ state prevents the corresponding feature from being distinctly visible in the absorption spectrum of **tBuCz3pTRZ**.

A simpler set of spectral features is observed in the emission spectra in toluene solution. All dendrimers exhibit a broad structureless CT_1_ emission (Figure 3a) and, in addition, the *meta*‐connection‐based dendrimers also show a high‐energy emission band, which is evident on a linear scale for **tBuCz2mTRZ** (Figure 3a) and on a logarithmic scale for the other *meta*‐connection dendrimers (Figure 3c). We attribute the broad CT emission to the CT_1_ state, as this band is symmetric to the CT_1_ absorption of all the dendrimers (Figure S4, Supporting Information). The CT_1_ emission spectra peak at (*λ*_PL_) 489 nm (2.54 eV), 493 nm (2.52 eV), 520 nm (2.38 eV), and 532 nm (2.33 eV) for **tBuCz3pTRZ**, **tBuCz3mTRZ**, **tBuCz2mTRZ**, and **tBuCz4mTRZ**, respectively. The emission spectrum of **tBuCz3pTRZ** matches with the one reported by Yamamoto et al.^[^ ^6e^ ^]^ The structured higher energy emission band is attributed to originating from the LE_1_ state, as it closely matches the dendron emission in energy and spectral shape (Figures 3c,d).

**Figure 4:**
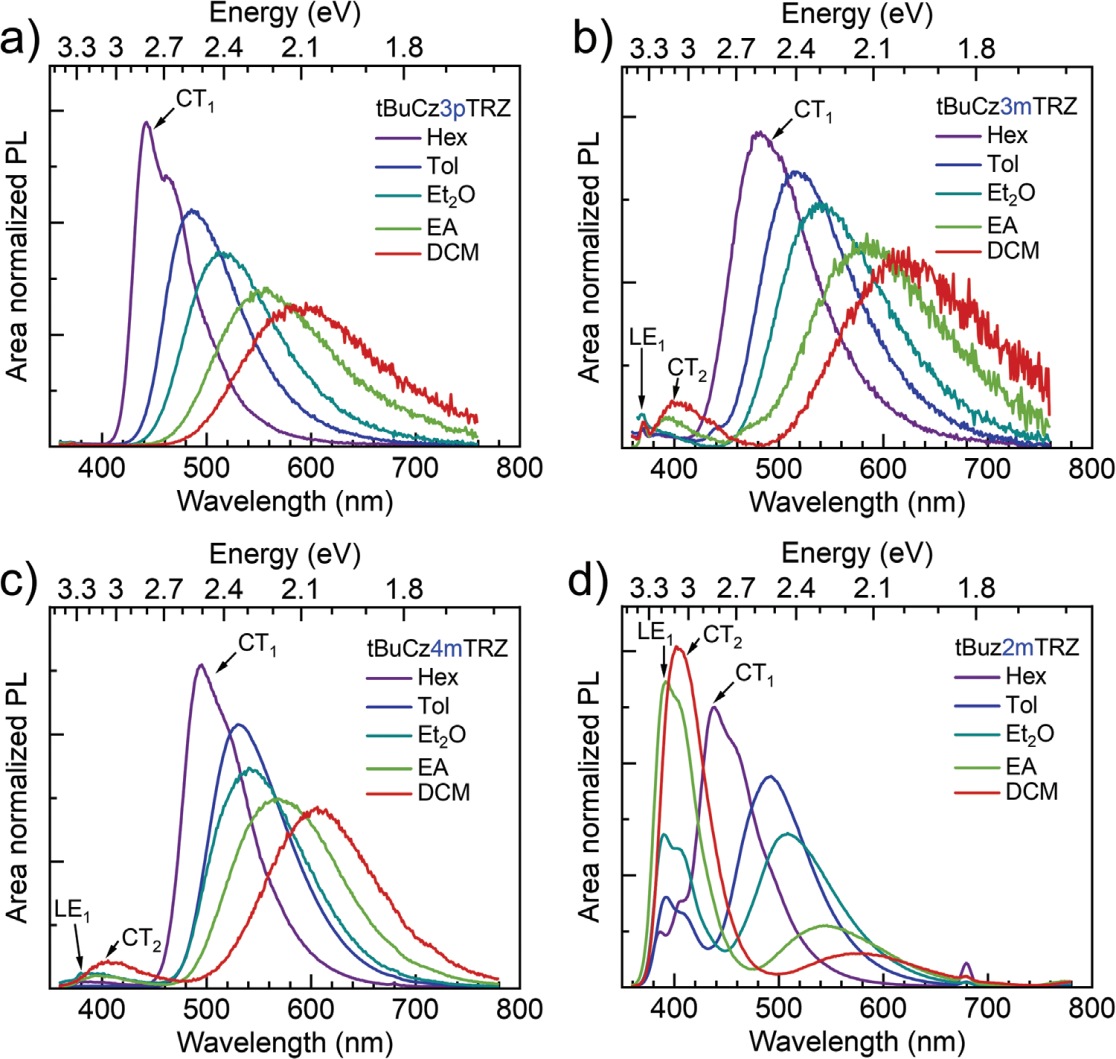
Solvatochromic PL spectra, normalized according to the integrated area (*λ*_exc_ = 340 nm; 3 × 10^−5^ m), of the dendrimers in hexane (Hex), toluene (Tol), diethyl ether (Et_2_O), ethyl acetate (EA) and dichloromethane (DCM). LE_1_ and CT_1_ refer to the lowest energy singlet LE and CT states and LE_X_ refers to the exciplex LE state as described in the text.

Solvatochromic PL measurements further confirm the nature of these states and explain the reason behind the observation of concomitant emission from different states. While only a single band is observed in **tBuCz3pTRZ**, three distinct bands can be identified in the emission spectra of the *meta*‐connection dendrimers in low polarity solvents (**Figure** **4**). Since the position of the high‐energy band (at around 380 nm/3.26 eV) in the *meta*‐connection dendrimers is insensitive to the solvent polarity it is readily assigned to a LE state, presumably LE_1_. The intensity of this LE band is extremely low in **tBuCz3mTRZ** and **tBuCz4mTRZ**; however, a strong LE signal is obtained in the emission spectra of **tBuCz2mTRZ,** even for high polarity solvents. The second band that changes only a little (about 120 meV) with solvent polarity is centered around 3 eV (400 nm). It is visible in Et_2_O, EtOAc, and CH_2_Cl_2_ (DCM) for **tBuCz3mTRZ** and **tBuCz4mTRZ**, and only in DCM for **tBuCz2mTRZ**. The lack of a pronounced solvatochromism also identifies it as a LE state. The position and the broad spectral shape are identical to the emission from a partial overlap excimer from a carbazole moiety,^[^ ^12^ ^]^ and an increased tendency to such excimer formation for more polar solvents is plausible. Thus, the features above 2.9 eV (420 nm) are attributed to an emission localized on the donor dendron and associated partial overlap excimers. In contrast to these LE features, with increasing solvent polarity we observe a bathochromic shift and a broadening of both the emission in **tBuCz3pTRZ** and the dominant low‐energy band in the *meta*‐connection dendrimers. We take this to confirm the charge‐transfer character of the low energy band already assigned to CT_1_.

Simultaneous emission from states of different energy can only be observed when the rate of internal conversion from the higher energy states to the lower energy states is sufficiently slow to compete with the rate of radiative decay of the higher energy states. Internal conversion from LE to CT state requires an electron transfer to the acceptor moiety, which occurs via an exchange mechanism (Dexter transfer) and thus depends strongly on the wavefunction overlap. Because of the more conjugated structure of **tBuCz3pTRZ**, this is expected to be fast in this case so that the only radiative contribution to the steady‐state emission spectrum comes from the lowest energy CT_1_ state, while the transfer rate is slowed down for the poorly conjugated *meta*‐connection dendrimers. The charge transfer rate decreases further with an increasing energy gap between the LE states (above 2.9 eV) and CT states (moving from about 2.8 eV down to about 2.1 eV with increasing solvent polarity) involved in these transitions. As a result, the rate of emission from the LE_1_ state and the associated excimer becomes comparable to the rate of internal conversion from LE_1_ to CT_1_, and thus, LE_1_ emission *and* CT_1_ emission can both be observed in the meta‐connection compounds.

### 2.3 Photophysics of Triplet Excited States and TADF

**Figure 5:**
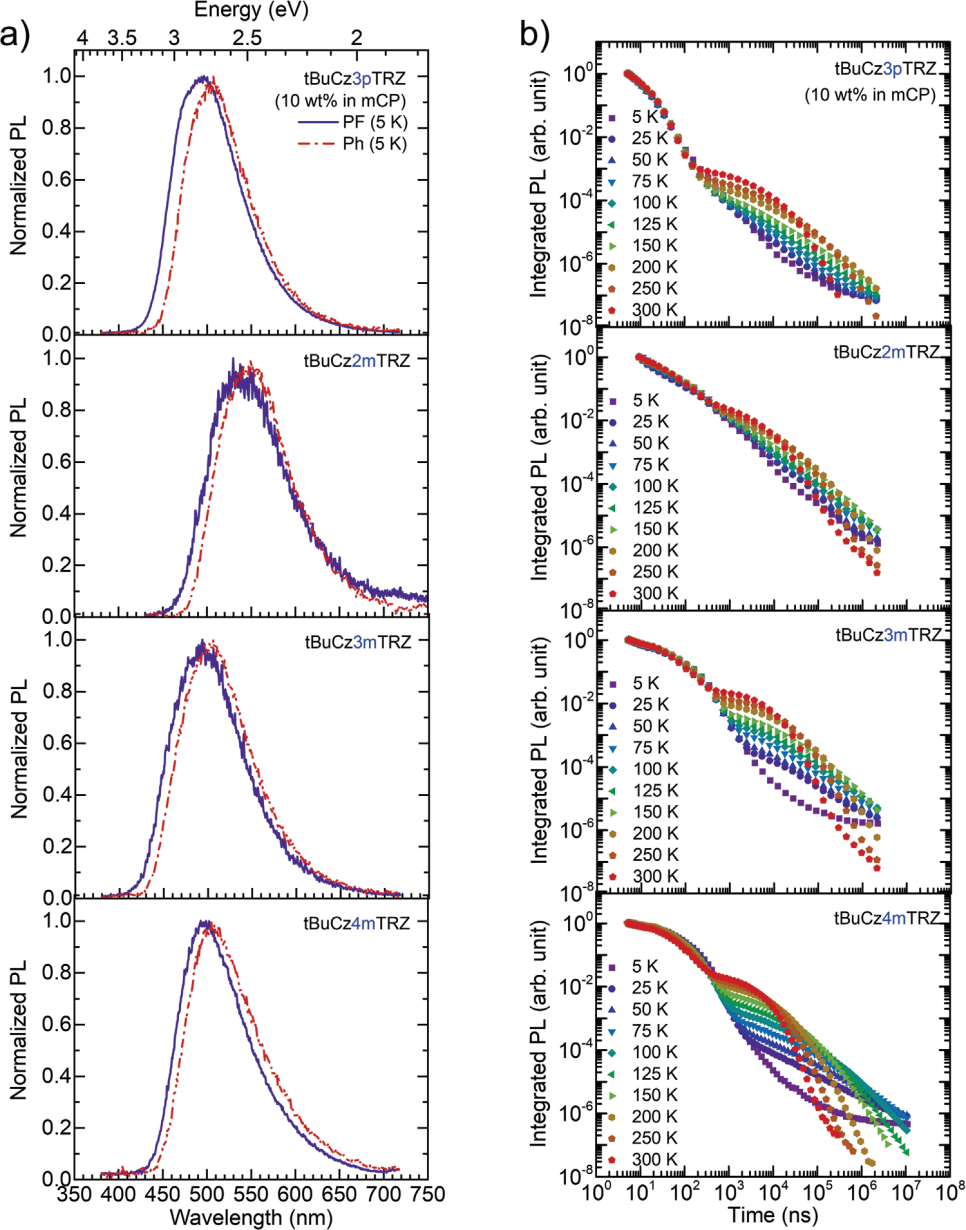
a) 5 K prompt fluorescence (delay time: 10 ns, blue solid line) and 5 K phosphorescence (delay time: 10 ms, red dashed‐dotted line) of the and b) temperature dependent PL decay curves for 10 wt% dendrimer films doped in mCP (*λ*_exc_ = 355 nm).

Having clarified the nature of the lowest singlet excited states, next we address the triplet states and the associated TADF properties of these dendrimers. For TADF materials, the energy difference between the lowest singlet (S_1_) and triplet excited states (T_1_), Δ*E*_ST_, is one of the key parameters used to assess the potential of the emitter to be used in OLEDs. We first investigated the solid‐state emission in a host matrix to reduce any impact of possible excimers (or aggregates) as well as the impact of bimolecular annihilation processes on the analysis of the monomolecular dendrimer properties such as quantum efficiencies and decay rate constants. **Figure** **5**a shows the spectra for the prompt fluorescence, PF (delay time: 10 ns, gating time: 7 ns) and phosphorescence, Ph (delay time: 10 ms, gating time: 1 ms) acquired in the doped films (10 wt% dendrimer in mCP) at 5 K. mCP was identified as a suitable host material because of its high‐triplet energy and good miscibility with various dopants. The characteristically broad and unstructured prompt fluorescence (phosphorescence) in the doped films indicates the dominant CT nature of the S_1_ (T_1_) state for each dendrimer as supported by our RT absorption and emission investigation probing the nature of the singlet state. The S_1_ and T_1_ energies are then taken from the onsets of PF and Ph, respectively, and are summarized in **Table** **1**. Accordingly, the Δ*E*_ST_ values are in the range of 50–100 meV. Similar S_1_, T_1_, and Δ*E*_ST_ values are also obtained for all the dendrimers in toluene glass at 5 K (Figure S5, Supporting Information). Such small differences between the lowest singlet and triplet excited states will facilitate a rapid RISC in these dendrimers, especially in the *meta*‐connection dendrimers where the Δ*E*_ST_ is smallest.

**Table 1:** Comparison of photophysical properties for 10 wt% dendrimer films doped in mCP

	S_1_ ^a)^ [nm eV]	T_1_ ^b)^ [nm eV]	Δ*E*_ST_ ^c)^ [meV]	*E*_act_ ^d)^ [meV]
**tBuCz3pTRZ**	436 (2.84)	452 (2.74)	100	52 ± 2
**tBuCz3mTRZ**	425 (2.92)	437 (2.84)	80	44 ± 3
**tBuCz2mTRZ**	470 (2.64)	482 (2.57)	70	22 ± 3
**tBuCz4mTRZ**	442 (2.81)	450 (2.76)	50	27 ± 2

^a)^ Lowest singlet energy determined from the onset of the PF (5 K; delay time 10 ns, gating time 7 ns);

^b)^ Lowest triplet energy determined from the onset of the Ph (5 K; delay time 10 ms, gating time 10 ms);

^c)^ Energy difference between S_1_ and T_1_;

^d)^ Activation energy determined from the Arrhenius plot of the DF intensity versus 1/*T*.

**Figure 6:**
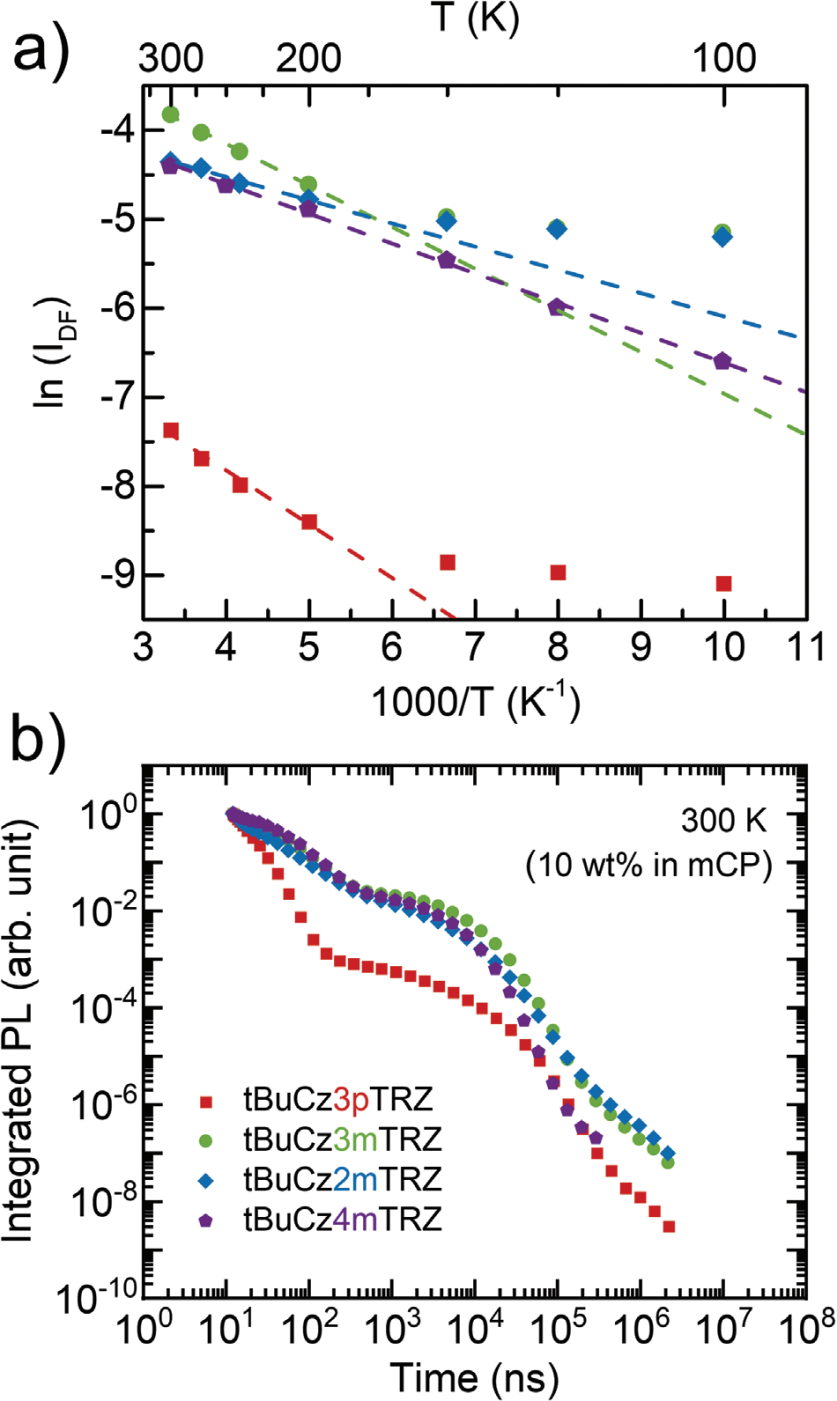
a) Arrhenius plot showing the variation of ln(*I*_DF_) with 1/*T* used to calculate *E*_act_ for the RISC process and b) 300 K PL decay curve for **tBuCz3pTRZ**, **tBuCz3mTRZ**, **tBuCz2mTRZ**, and **tBuCz4mTRZ** doped films (10 wt% in mCP).

Figure 5b shows the photoluminescence (PL) decays of the 10 wt% doped dendrimer films in mCP. The PL decays are typical for a TADF emitter, i.e., they are characterized by two different regimes that correspond to prompt fluorescence and delayed fluorescence, and the intensity of DF increases with the increasing temperature. Notably, for **tBuCz3mTRZ** and **tBuCz4mTRZ**, much stronger thermal activation is observed between 5 K and 25 K as compared to the thermal activation at higher temperatures, implying extremely efficient RISC as it is an endothermic process. To calculate the effective activation energy barrier (*E*_act_) for the RISC process, the intensity of the DF (at a delay time of 1 µs) is plotted logarithmically against 1000/*T*, as shown in **Figure** **6**a. A linear relationship of this Arrhenius plot above 200 K indicates the absence of phosphorescence within this temperature range. The *E*_act_ values (Table 1) thus obtained are approximately half the corresponding Δ*E*_ST_ values. For comparison, the PL decays at 300 K are plotted in Figure 6b. The PF decay lifetime of **tBuCz3pTRZ** is shorter than those of the *meta*‐connection dendrimers owing to the greater hole‐electron overlap of the lowest ^1^CT state, while the DF/PF ratio is higher for the *meta*‐connection dendrimers. The PL decays at 300 K along with the photoluminescence quantum yield (PLQY) values were used to calculate the quantum efficiencies and rate constants associated with different kinetics processes resulting in the excited state of the 10 wt% doped films in mCP. The parameters were determined according to the procedure described in the Supporting Information and are summarized in **Table** **2**. Of particular interest are the total PLQY values (*φ*_PL_), the quantum efficiency for DF (*φ*_DF_), and the RISC rates (*k*_RISC_). Under N_2_, the **tBuCz3pTRZ** film has the highest PLQY of 89% while **tBuCz3mTRZ** and **tBuCz4mTRZ** have similar *φ*_PL_ of about 80% and **tBuCz2mTRZ** has the lowest *φ*_PL_ of 65%. Interestingly, *φ*_DF_ is significantly lower for **tBuCz3pTRZ** (0.19) when compared to the *meta*‐connection dendrimers (0.63, 0.45, and 0.50 for **tBuCz3mTRZ**, **tBuCz2mTRZ,** and **tBuCz4mTRZ**, respectively). This illustrates that although the *φ*_PL_ of **tBuCz3pTRZ** is the highest, most of the contribution to the PL comes from PF and not from DF, i.e., from the photoexcited singlets and not the RISC‐activated singlets. Furthermore, the RISC efficiency (*φ*_RISC_) and RISC rate (*k*_RISC_) of **tBuCz3pTRZ** are also significantly lower when compared to the *meta*‐connection dendrimers (for values, refer Table 2). These parameters are of crucial importance to the device performance because they govern to population of triplet excitons because of electrical excitation. In view of the device application, we note the remarkable efficiency of **tBuCz3mTRZ** for RISC of 95% in the doped film, which remains high at 89% in the neat film.

**Table 2:** Comparison of photophysical properties in 10 wt% dendrimer films doped in mCP and neat dendrimer films

	*τ*_PF_ ^a)^ [ns]	*τ*_DF_ ^b)^ [µs]	*φ*_PL_ ^c)^ [%]	*φ*_DF_/*φ*_PF_ ^d)^	*φ*_PF_ ^e)^	*φ*_DF_ ^f)^	*φ*_ISC_ ^g)^	*φ*_RISC_ ^h)^	*k*_F_ ^i)^ [×10^7^ s^−1^]	*k*_ISC_ ^j)^ [×10^7^ s^−1^]	*k*_RISC_ ^k)^ [×10^5^ s^−1^]	$k_{\mathrm{NR}}^{T}$ ^l)^ [×10^5^ s^−1^]
Doped film (10 wt% dendrimer in mCP)												
**tBuCz3pTRZ**	9.8	3.1	89	0.27	0.70	0.19	0.71	0.71	7.19	3.05	0.5	0.2
**tBuCz3mTRZ**	50.2	5.5	81	3.51	0.18	0.63	0.95	0.95	0.36	1.63	3.7	0.2
**tBuCz2mTRZ**	40.3	4.0	65	2.31	0.20	0.45	0.80	0.87	0.49	1.99	2.2	0.3
**tBuCz4mTRZ**	52.2	3.4	79	1.68	0.29	0.50	0.71	0.89	0.57	1.35	3.5	0.4
Neat dendrimer film												
**tBuCz3pTRZ**	13.4	1.4	51	0.56	0.33	0.18	0.67	0.53	2.44	5.04	0.7	1.4
**tBuCz3mTRZ**	54.2	2.2	62	3.04	0.15	0.47	0.85	0.89	0.28	1.56	6.5	0.7
**tBuCz2mTRZ**	96.7	1.1	59	0.85	0.32	0.27	0.68	0.67	0.33	0.70	5.4	1.9
**tBuCz4mTRZ**	62.7	1.7	67	1.2	0.31	0.37	0.70	0.78	0.49	1.11	4.3	0.9

^a)^ Lifetime of prompt emission (obtained by single exponential fitting of prompt emission decay regime at RT) *λ*_exc_ = 355 nm;

^b)^ Average lifetime of delayed emission, $\tau_{\mathrm{DF}}=\frac{\int t\;I_{\mathrm{DF}}\;dt}{\int I_{\mathrm{DF}}\;dt}$;

^c)^ Photoluminescence quantum yield, under N_2_;

^d)^ DF/PF = ∫$\frac{I_{\mathrm{DF}(t)}}{I_{\mathrm{PF}(t)}}dt$;

^e)^ PF quantum efficiency;

^f)^ DF quantum efficiency;

^g)^ ISC quantum efficiency;

^h)^ RISC quantum efficiency;

^i)^ Radiative decay rate of singlet excitons;

^j)^ ISC rate;

^k)^ RISC rate;

^l)^ Nonradiative rate of triplet excitons. The procedure of determining all the photophysical parameters is described in Section S1 of the Supporting Information.

For the photophysical character in neat films under N_2_, a significant decrease in *φ*_PL_ is observed for **tBuCz3pTRZ** (51% in neat film compared with 89% in doped film), while in contrast, the concentration quenching of **tBuCz3mTRZ** (62% in neat film compared with 81% in doped film), **tBuCz2mTRZ** (59% in neat film compared with 65% in doped film) and **tBuCz4mTRZ** (67% in neat film compared with 79% in doped film) is suppressed. The PL decays of the neat films also reveal the temperature dependence of the DF as low as 25 K, implying that efficient RISC behavior is preserved in neat films (Figure S7, Supporting Information). Rate constants determined from the RT PL decays (Table 2), though affected by the presence of bimolecular processes (not considered in the analysis described in the Supporting Information), resemble qualitatively to the ones derived for the 10 wt% doped dendrimer films. This implies that the conclusions derived for the devices based on doped OLEDs, based on the photophysical parameters (*φ*_DF_, *φ*_RISC_, and *k_RISC_*), remain valid for neat film OLEDs as well.

## 3 Quantum Chemical Calculations

The photophysical study reveals that the substitution pattern of the donor dendrons has a significant impact on the photophysics of the dendrimers. The *meta*‐connection dendrimers possess the smaller Δ*E*_ST_, show more efficient TADF with faster RISC, and thus the corresponding OLEDs exhibit improved performance. However, to understand the factors governing the differences in TADF properties and how these correlates with the donor dendron substitution pattern, we first must understand both the nature and degree of mixing of the low‐lying excited states.

It has been advanced that the vibrational coupling between closely lying triplet excited states can assist the RISC process via a second order perturbation effect.^[^ ^13^ ^]^ To verify the implication of higher lying triplet states in the RISC process, we performed excited state geometry optimizations for the model dendrimers **tBuCz1pTRZ** and **tBuCz1mTRZ** (Figures S14 and S15, Supporting Information) that contain only one donor dendron connected to the triphenyl triazine unit instead of three. We found that the optimized T_2_ state lies considerably higher in energy than the T_1_ state ($\Delta E_{T_{2}-T_{1}}$ = 370 meV for **tBuCz1pTRZ** and 220 meV for **tBuCz1mTRZ**). We note that the additional dendrons in **tBuCz3pTRZ** and **tBuCz3mTRZ** can lead to the introduction of additional degenerate triplet states; however, this will not affect our conclusion that T_2_ (or indeed any other higher lying triplet state) is expected to be only minimally involved in the RISC process. Thus, the explanation of the observed difference in the RISC rates warrants consideration within the limit of first order perturbation theory.

*k*_RISC_ can be computed in the framework of Fermi's Golden rule^[^ ^14^ ^]^

(1) $$k_{\mathrm{RISC}}=\frac{2\pi }{\hbar }\;\rho_{\mathrm{FC}}{\left|\langle S_{1}|{\hat{H}}_{\mathrm{SO}}|T_{1}\rangle \right|}^{2}$$

where $\left\langle S_{1}\left|{\hat{H}}_{\mathrm{SO}}\right|T_{1}\right\rangle$ is the spin orbit coupling (SOC) matrix element from T_1_ to S_1_ and *ρ*_FC_ denotes the Franck–Condon‐weighted density of states term, which can be evaluated according to the semi‐classical Marcus–Hush formulation^[^ ^15^ ^]^

(2) $$\rho_{\mathrm{FC}}=\frac{1}{\sqrt{4\pi \lambda k_{B}T}}\;\exp\left[-\frac{{\left(\Delta E_{\mathrm{ST}}+\lambda \right)}^{2}}{4\lambda k_{B}T}\right]$$

*λ*  = *λ*_inter_  + *λ*_intra_ is the Marcus reorganization energy (Figure S16, Supporting Information) associated with the vibrational coupling to intermolecular and intramolecular low frequency vibrational modes. Equation (2) assumes that the quantum nature of the low frequency modes (with energies, ℏ*ω*, of a few meV) does not need to be considered explicitly, i.e., *k_B_T*  ≫  ℏ*ω*, and can be treated classically.^[^ ^15^, ^16^ ^]^ Thus, *k*_RISC_ depends on the adiabatic Δ*E*_ST_, the SOC matrix element in the geometry of the relaxed T_1_ state and the coupling to the low frequency modes associated with the RISC from T_1_ to S_1_. We have used **Cz3pTRZ** and **Cz3mTRZ** as model compounds to evaluate these quantities at the TDA‐DFT M06‐2X/6‐31G(d,p) level. The *tert*‐butyl (tBu) groups were replaced with hydrogen atoms in order to reduce the computational cost.

**Figure 7:**
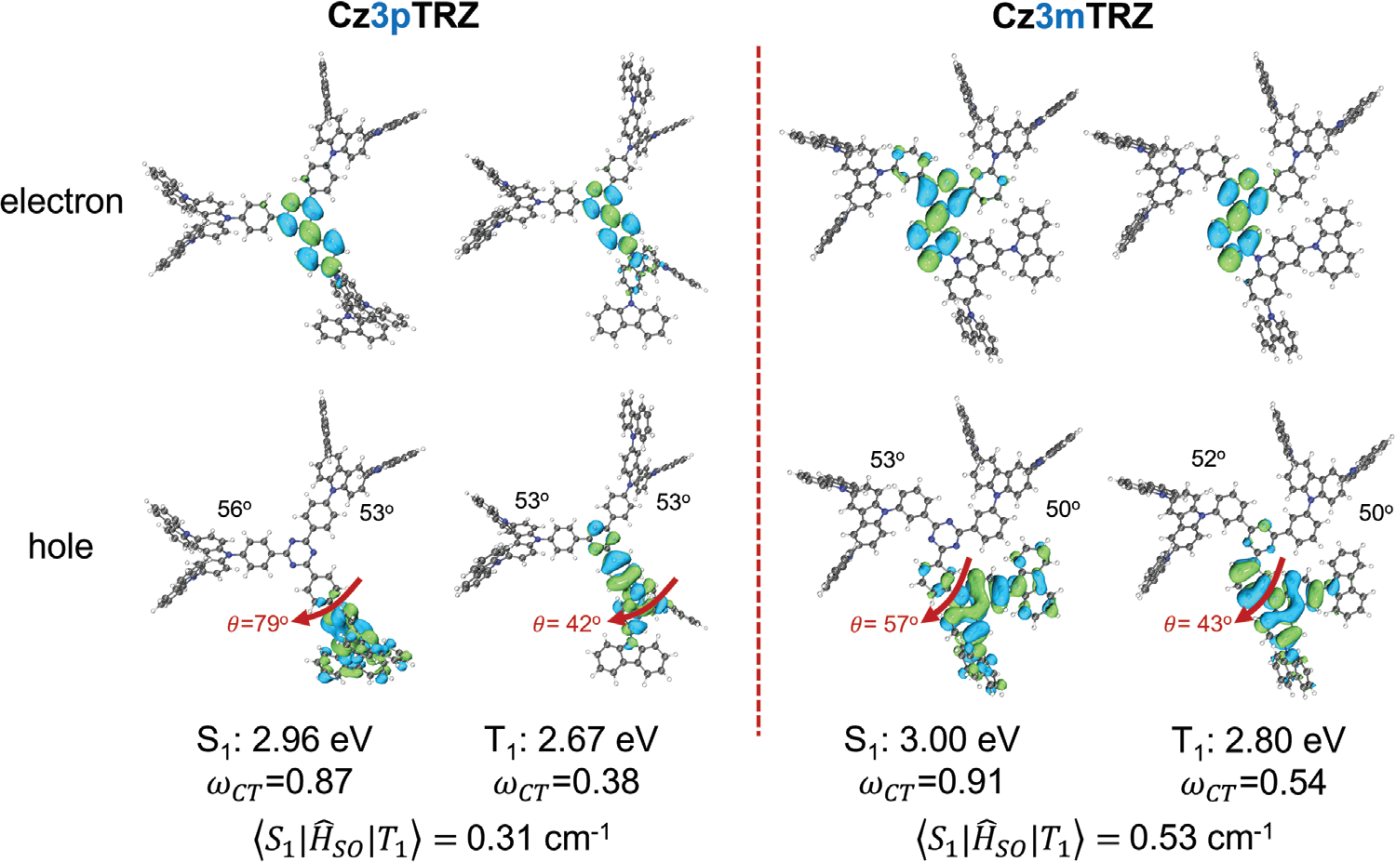
Natural transition orbital (NTO) pairs for the S_1_ and T_1_ states of **Cz3pTRZ** and **Cz3mTRZ**. The spin–orbit coupling (SOC) matrix elements are also provided.

In the S_1_ state equilibrium geometry of **Cz3pTRZ** (**Figure** **7**) the hole and electron natural transition orbitals (NTOs) are localized on the donor dendron and phenyltriazine, respectively. Additionally, the donor dendron adopts a highly twisted conformation with respect to the TRZ with a dihedral angle of 79°. This indicates that the S_1_ state possesses intramolecular CT character, which is also confirmed by a charge transfer number (*ω*_CT_) value of 0.87; *ω*_CT_ (see the Experimental Section for the definition) takes values between 0 (for a Frenkel exciton) and 1 (for a pure CT exciton). The S_1_ state of **Cz3mTRZ** possesses a similar CT character (*ω*_*CT*_ = 0.91) but adopts a less twisted conformation with a much smaller dihedral angle, *θ* = 57° as compared to that of **Cz3pTRZ** in its ^1^CT state. While the electron NTO in the T_1_ state of **Cz3mTRZ** is localized on the phenyltriazine (while some delocalization to the carbazole is found for **Cz3pTRZ**), the hole NTO extends from the central carbazole of the donor dendron to the bridging phenylene and to a smaller extent to the triazine unit (unlike **Cz3pTRZ** where there is a larger amount of hole density on the triazine unit). This leads to a greater CT character in the T_1_ state of **Cz3mTRZ** (*ω*_CT_ = 0.54) as compared to **Cz3pTRZ** (*ω*_CT_ = 0.38), even though *θ* remains the same (42°–43°). It is worth emphasizing at this point that while the RISC process in **Cz3pTRZ** involves a relatively large dihedral angle change (Δ*θ*) of about 37°, there is a much more moderate change of 13° in **Cz3mTRZ**. The adiabatic Δ*E*_ST_ for **Cz3pTRZ** (Δ*E*_ST_ = 290 meV; S_1_ = 2.96 eV, T_1_ = 2.67 eV) is thus larger than that for **Cz3mTRZ** (Δ*E*_ST_ = 200 meV; S_1_ = 3.00 eV, T_1_ = 2.80 eV), in qualitative agreement with the experimentally determined singlet–triplet gaps; however, the computed Δ*E*_ST_ values are larger than the experimental values due to the fact that the M06‐2X functional overestimates the singlet state energies.

Experimentally, it was found that the adiabatic Δ*E*_ST_ for **tBuCz3mTRZ** (80 meV) is smaller than that of **tBuCz3pTRZ** (100 meV). According to the Boltzmann distribution, this Δ*E*_ST_ difference can lead to a 40% (1.4 times) enhancement of RISC in **tBuCz3mTRZ** as compared to **tBuCz3pTRZ** and thus, the Δ*E*_ST_ difference alone is not sufficient to explain the observed differences in *k*_RISC_ for *meta‐* versus *para‐*linked dendrimers. However, when the Δ*E*_ST_ values are similar, even a few tenths of cm^−1^ increase in the SOC matrix element can lead to a considerable enhancement of the RISC rate.^[^ ^14^ ^]^ We thus calculated the SOC matrix element for the RISC process $\left(\left\langle S_{1}|{\hat{H}}_{\mathrm{SO}}|T_{1}\right\rangle \right)$ at the T_1_ state geometry, since the initial state for the RISC process is the T_1_ state. The calculated SOC matrix element was found to be higher for **Cz3mTRZ** (0.53 cm^−1^) as compared to **Cz3pTRZ** (0.31 cm^−1^). Closer consideration of the NTOs at the optimized triplet excited state geometries reveals that for **Cz3mTRZ** there is a change in the orbital character only for the HOMO NTO between S_1_ and T_1_ due to the reduced conjugation present in **Cz3mTRZ** (while for **Cz3pTRZ** there is a change for both the HOMO and LUMO NTOs), and according to El‐Sayed's rule this will lead to larger SOC in **Cz3mTRZ**.

The coupling to the vibrational modes approximated in terms of the Marcus reorganization energy is also an important parameter, as it controls the Franck–Condon‐weighted density of states (Equation (2)) and hence the *k*_RISC_ (Equation (1)). The calculated intramolecular (*λ*_intra_) reorganization energy for **Cz3pTRZ** (275 meV) was found to be higher as compared to **Cz3mTRZ** (155 meV). We recall that the S_1_ state for both *para*‐ and *meta*‐linked dendrimers is strongly CT in nature and that there is a nearly orthogonal (i.e., *θ* =  79°) conformation adopted between the donor dendron and the phenylenes. While geometric relaxation from this is restricted in **Cz3mTRZ** because of steric constraints, it is possible for there to be significantly larger changes in dihedral angles in **Cz3pTRZ**. This implies a larger reorganization energy for the T_1_ → S_1_ transition in **Cz3pTRZ** as compared to **Cz3mTRZ**.

As a final check, the RISC rates can be calculated within the framework of Equations (1) and (2) using the computed reorganization energies and SOC values along with the experimentally determined Δ*E*_ST_ values (see Table S5 in the Supporting Information). Ignoring the outer sphere contribution of the surrounding medium to the reorganization energy, the *k*_RISC_ values are 5.1 × 10^5^ s^−1^ for **Cz3pTRZ** and 9.4 × 10^6^ s^−1^ for **Cz3mTRZ**. By considering an intermolecular reorganization energy (*λ*_inter_) value of 300 meV for both **Cz3pTRZ** and **Cz3mTRZ**, this reduces *k*_RISC_ to 0.2 × 10^5^ s^−1^ for **Cz3pTRZ** and 3.6 × 10^6^ s^−1^ for **Cz3mTRZ**, values that are in excellent agreement with those experimentally determined for **tBuCz3pTRZ** and **tBuCz3mTRZ**. Thus, the smaller Δ*E*_ST_, greater SOC and smaller reorganization energy combine to lead to a faster *k*_RISC_ in **tBuCz3mTRZ** compared to **tBuCz3pTRZ**.

## 4 Discussion

Based on the photophysical analysis we can conclude that all dendrimers in the doped and neat films exhibit high PLQY. However, the contribution of delayed emission to the total emission and the RISC rate, both being the characteristic of an efficient RISC process and of key importance to OLED operation, are much greater/faster in the case of the *meta*‐connected dendrimers. This is consistent with the improved EL performance of the *meta*‐dendrimer OLEDs. The ISC rate constant (*k*_ISC_) is approximately similar across the series of dendrimers, with slightly lower values for *meta*‐connected dendrimers; however, *k*_RISC_ is several times faster for the *meta*‐connected dendrimers. Though this is the first report where *meta*‐connected dendrimers have been reported, the impact of the substitution pattern of donors with respect to the acceptor on the TADF properties has been studied extensively for small molecules.^[^ ^9^ ^]^ The OLEDs employing the *meta*‐isomers often exhibit higher EQEs than the devices with the *para*‐isomer.^[^ ^17^ ^]^ This improvement has been ascribed to the reduced conjugation between donor and acceptor units and thus reduced Δ*E*_ST_. Recently, we also underlined the role of *meta*‐linkage in reducing the conjugation between the donor and acceptor units.^[^ ^18^ ^]^

In the present work as well, the extinction coefficient of the ^1^CT state decreases by an order of magnitude when the more conjugated *para*‐connection is replaced by the less conjugated *meta*‐connection (Figure 3b). This is also evident from the anti‐Kasha emission in *meta*‐dendrimers (Figure 3c) arising from the slow charge transfer from the ^1^LE to the ^1^CT state owing to the weaker conjugation in *meta*‐connected dendrimers as compared to the *para*‐connected dendrimer. However, the reduction in conjugation and the associated decrease in Δ*E*_ST_ alone cannot explain the increase in *k*_RISC_ rate in going from *para*‐ to *meta*‐connected dendrimers. Penfold, Monkman and coworkers advanced that the nonadiabatic (vibronic) coupling between a ^3^CT state and a higher‐lying ^3^LE state can promote RISC to the ^1^CT state.^[^ ^13^ ^]^ However, this mechanism is not operational in the dendrimers in this study as these LE T_n_ states are too destabilized to be implicated in any significant fashion to the RISC process.

Nevertheless, it is worth recalling that there are other factors within the first order perturbation theory (Equations (1) and (2)) that can be considered in the chemical design other than Δ*E*_ST_, these include the SOC matrix element for the RISC process $\left(\left\langle S_{1}|{\hat{H}}_{\mathrm{SO}}|T_{1}\right\rangle \right)$ and the reorganization energy (*λ*). Though the molecular design of donor‐acceptor TADF emitters has most often focused on either the minimization the Δ*E*_ST_ or the enhancement of the SOC for the T_1_→S_1_ transition, the role of *λ* has been frequently overlooked. Furthermore, from a chemical design perspective, optimizing these parameters (Δ*E*_ST_, SOC, and *λ*) should be easier than optimizing the *T_n_* − *T*_1_ gap. Increasing RISC is the central issue for TADF emitters and, as discussed above, *meta*‐connection dendrimers possess several design advantages over *para*‐connection dendrimer with respect to enhancing RISC. We emphasize that the change in geometry between T_1_ and S_1_ is an important parameter, especially in the case of bulky dendrimer systems and can be used as a general design principle.

**Figure 8:**
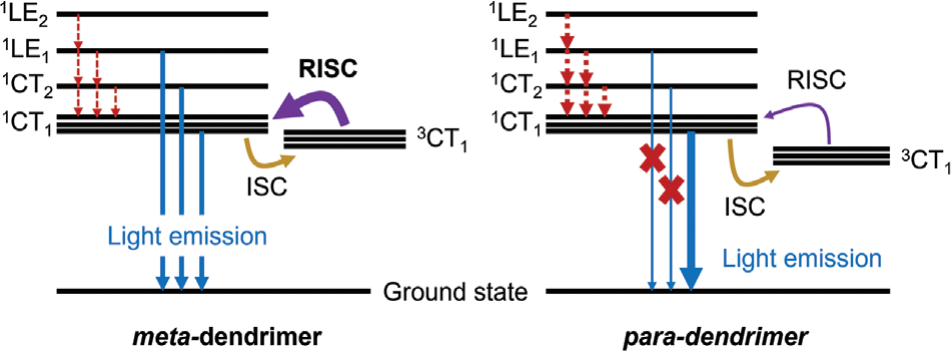
Jablonski diagram describing the dual CT/LE emission (blue solid), internal conversion (red dot), intersystem crossing (yellow solid), reverse intersystem crossing (purple solid) processes for *meta*‐/*para*‐dendrimers. The thicker arrow lines indicate a faster rate of corresponding process while thinner arrow lines indicate a slower rate of the corresponding process.

The results inspired us to further improve the design of TADF dendrimers in related study^[^ ^10^ ^]^ by combining the advantages of *para*‐ and *meta*‐connected dendrimers as illustrated in **Figure** **8**. Through the introduction of both *para*‐ and meta‐connected donor dendrons about the triazine acceptor, the dendrimer **tBuCz2m2pTRZ** was then found to not only inherit a high RISC rate, large oscillator strength, but also shows a desired suppressed concentration quenching as evident by the improved OLED performance.^[^ ^10^ ^]^

## 5 Conclusions

In summary, highly efficient TADF dendrimers were developed with a strategy of weak conjugation between the multiple donor dendrons and the TRZ acceptor. This design affords an extremely efficient utilization of triplet excitons because of a vanishing singlet–triplet splitting energy, which is critical to determine the efficiency of TADF materials. From our thorough investigation of the dendrimer photophysical properties in toluene solution, we were able to reveal the origin and nature of the lowest excited states. Investigations in the solid state revealed that better TADF properties are obtained for the *meta*‐linked dendrimers when compared to the *para*‐linked dendrimers, thus demonstrating the validity of this design strategy for a highly efficient TADF dendrimer by combining the feature of multiple donors, weak conjugated connection, and dendritic structure. Although both *meta*‐ and *para*‐linked dendrimers possess small Δ*E*_ST_ values, normally implying similar *k*_RISC_ given the similar molecular structures, what the present study reveals is that the reorganization energy can play an important role in enhancing *k*_RISC_, and that this can be modulated as a function of the donor dendron substitution pattern about the central triazine acceptor.

## 6 Experimental Section

### Synthesis

The procedures for the synthesis of the TADF dendrimer and the corresponding characterization are reported in the Supporting Information.

### Theoretical Calculations

The ground state geometries of the para and meta‐connected compounds were optimized in the gas phase using density functional theory at the M06‐2X/6‐31G(d,p) level of theory.^[^ ^19^ ^]^ Subsequently, the first singlet and triplet excited state geometries were optimized through linear response time‐dependent density functional theory at the M06‐2X/6‐31G(d,p) level within the Tamm–Dancoff approximation (TDA).^[^ ^20^ ^]^ It was found previously that the M06‐2X exchange‐correlation functional provides a good description of the excited states in carbazole‐triazine bipolar host compounds.^[^ ^18^ ^]^ Calculations were performed using the Gaussian 09 software.^[^ ^21^ ^]^ Electron–hole natural transition orbitals were computed using the Multiwfn software.^[^ ^22^ ^]^ The nature of the charge‐transfer character of the transitions was further quantified in terms of charge transfer numbers *ω*_CT_ computed by a transition density matrix analysis^[^ ^23^ ^]^ based on a defragmentation of the molecules in donor and acceptor units. Internal, structural reorganization energies *λ* of the compounds for the reverse intersystem crossing T_1_→ S_1_ were computed by evaluating the total energy difference between the singlet state at the optimized T_1_ geometry and the singlet state at the optimized S_1_ geometry, i.e., *λ* = *E*_S1_(T_1_) − *E*_S1_(S_1_).

The spin–orbit coupling elements for reverse intersystem crossing between the lowest triplet (T_1_) and singlet (S_1_) electronic states were computed at the relaxed T_1_ geometries within the TDA by employing the one‐electron Breit Pauli Hamiltonian. Spin–orbit coupling calculations were performed using the Q‐Chem 5.2 software.^[^ ^24^ ^]^

### Photophysical Characterization

For steady‐state emission studies, degassed solutions were prepared via three freeze–pump–thaw cycles and spectra were taken using home‐made Schlenk quartz cuvette. Steady‐state spectra were recorded at room temperature using an Edinburgh Instruments F980 fluorimeter. Samples were excited at 340 nm for steady‐state measurements.

For time‐resolved PL measurements, the solution was prepared by dissolving the dendrimers into toluene at a concentration of 3 × 10^−5^ m, then heated and sonicated the solution before measurement. Thin film was prepared by spin‐coating a solution of 10 wt% dendrimer in mCP at 2000 rpm on a quartz substrate. The solution for spin‐coating was prepared by dissolving the dendrimer and mCP in chloroform, and then filtered using a syringe filter. The time‐resolved PL measurements of the thin films and solutions were carried out using an iCCD camera by exponentially increasing delay and gating times where gating time is kept lower by 10 times compared to the delay time. The samples and cuvettes were kept in a continuous flow He‐cryostat with temperature controller. They were excited at 355 nm by a lamp‐pumped Nd:YAG laser (Innolas SpitLight 600). Emission from the samples was focused onto a spectrograph (Oriel MS257) and detected on a gated iCCD camera (iStar A‐DH334T‐18F‐03). The measurements were recorded under He exchange gas unless otherwise stated. Solution PLQY measurements were obtained using a Jasco FP‐8600 spectrofluorometer for excitation at 325 nm.

### OLED Fabrication

The OLED devices were fabricated in bottom‐emitting architecture. A prepatterned indium tin oxide (ITO) glass substrate was used as the anode. PEDOT: PSS8000 was spin‐coated on the clean ITO substrate as the hole‐injection layer and then thermally annealed at 140 °C for 1 h before transferred to a glovebox. The emitting layer (EML) was formed by spin‐coating the dendrimer from chlorobenzene solution with a concentration of 10 mg mL^−1^. TmPyPB, LiF and Al were then vacuum‐deposited on EML subsequently in a vacuum chamber. Detailed operation and characterization are reported in the Supporting Information.

## Conflict of Interest

Dr. Dianming Sun and Prof. Eli Zysman‐Colman are the co‐inventors of a patent, PCT/GB2021/052844, based on the materials in this manuscript.

## Supporting information

Supporting Information

## Data Availability

The research data supporting this publication can be accessed at https://doi.org/10.17630/1c0258ff-02d5-4332-a0a3-6acdc0892eb2.
