# Supplementary material for: Regiochemistry of Donor Dendrons Controls the Performance of Thermally Activated Delayed Fluorescence Dendrimer Emitters for High Efficiency Solution‐Processed Organic Light‐Emitting Diodes
Source: Adv Sci (Weinh). 2022 Apr 25;9(20):2201470. doi: 10.1002/advs.202201470 (PMC9284163; doi:10.1002/advs.202201470)
Supplement: Supplementary file 1 — Supporting Information [file ADVS-9-2201470-s001.pdf]

Supplementary Information

Regiochemistry of Donor Dendrons Controls the Performance of Thermally Activated Delayed Fluorescence Dendrimer Emitters Translating in Their Use in High Efficiency Solution-Processed Organic Light-Emitting Diodes

Dianming Sun[1,2†], Rishabh Saxena[3†], Xiaochun Fan[1], Stavros Athanasopoulos[5], Eimantas Duda[3], Ming Zhang[1], Sergey Bagnich[3], Xiaohong Zhang[1*], Eli Zysman-Colman[2*] and Anna Köhler[3,4*]*

Table of Contents

Synthesis and Characterization 3

Electrochemistry measurements.. 7

OLED fabrication and characterization 10

Photophysical characterization.. 13

Determination of photophysical rate constants.. 18

Theoretical calculations... 19

NMR spectra.. 33

Reference 45

Synthesis and Characterization

The starting material, **G2tBuCzH** and 2,4,6-tris(3-bromophenyl)-1,3,5-triazine, were synthesized according to literature procedures.[1] All solvents and reagents were obtained from commercial sources and used as received. Air-sensitive reactions were performed under a nitrogen atmosphere using Schlenk techniques, no special precautions were taken to exclude air or moisture during work-up. Anhydrous THF was obtained from a Pure Solv™ solvent purification system (Innovative Technologies). Flash column chromatography was carried out using silica gel (Silia-P from Silicycle, 60 Å, 40-63 µm). Analytical thin-layer-chromatography (TLC) was performed with silica plates with aluminium backings (250 µm with F-254 indicator). TLC visualization was accomplished by 254/365 nm UV lamp. ^{1}H, ^{13}C, ^{19}F and 2D NMR spectra were recorded on a Bruker Advance spectrometer. ^{19}F NMR spectrum was recorded with proton decoupling. ^{1}H and ^{13}C NMR spectra were referenced residual solvent peaks with respect to TMS (δ = 0 ppm). The following abbreviations have been used for multiplicity assignments: "s" for singlet, "d" for doublet, "t" for triplet, "m" for multiplet, and "brs" for broad singlet. Melting points were measured using open-ended capillaries on an Electrothermal melting point apparatus IA9200 and are uncorrected. Matrix Assisted Laser Desorption/Ionization-Time of Flight-Mass Spectrometry (MALDI-TOF-MS) was performed by EPSRC National Mass Spectrometry Service Centre (NMSSC), Swansea. Elemental analyses were performed by Mr. Stephen Boyer, London Metropolitan University. tBuCz3pTRZ was synthesized according to the procedure in the literature.[1a]

Detailed synthetic routes and procedures of the target compounds are outlined as below.

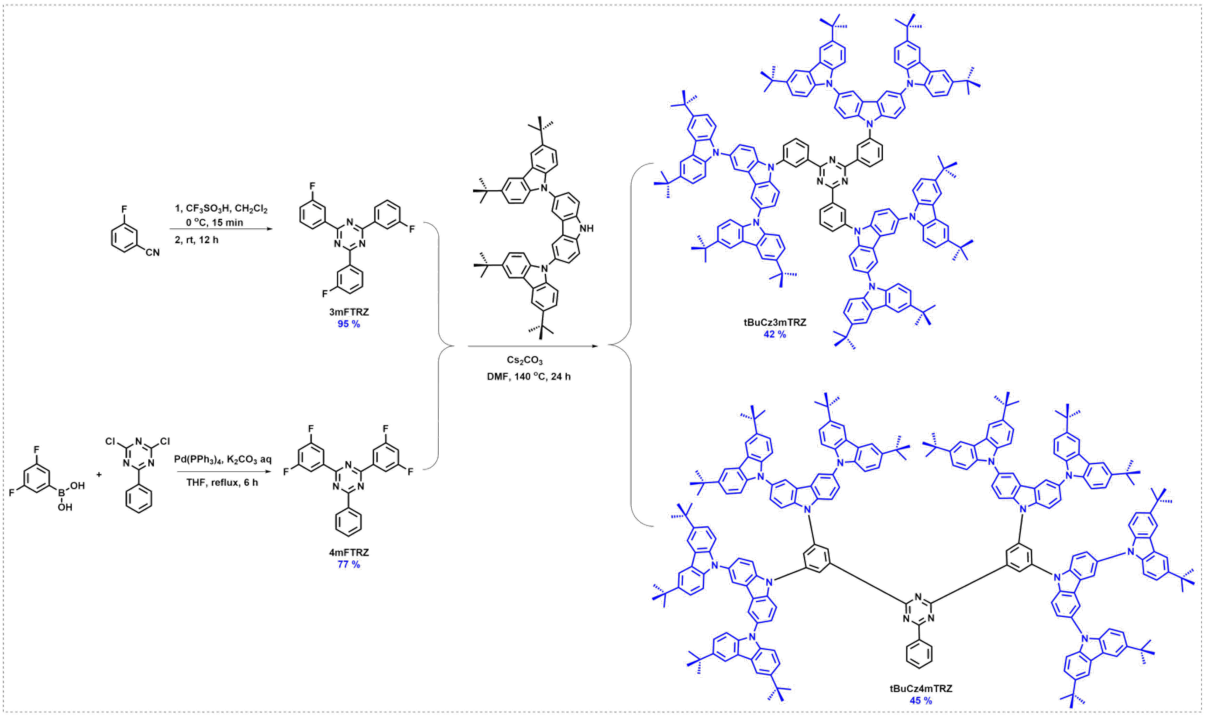

Scheme S1. Synthetic route for **tBuCz3mTRZ** and **tBuCz4mTRZ**.

Synthesis of *2,4-bis(3,5-difluorophenyl)-6-phenyl-1,3,5-triazine* (**4mFTRZ**).

A mixture of (3,5-difluorophenyl)boronic acid (632 mg, 4 mmol, 2 equiv.), 2,4-dichloro-6-phenyl-1,3,5-triazine (452 mg, 2 mmol, 1 equiv.) and tetrakis(triphenylphosphine)palladium(0) (115 mg, 0.1 mmol, 0.25 equiv.) in degassed THF (20 mL), and 2M K_2CO_3 (5 mL) aqueous solution was refluxed for 12 h. After cooling to room temperature, the resulting precipitate was filtered and washed with both THF and DCM (each 3×20 mL). Then the collected precipitate was dried in a desiccator to afford a white solid (590 mg). **Yield**: 77%. **R*f***: 0.45 (Hexane: DCM=2:1 on silica plate). **Mp**: decomposes before melting. **^{1}H NMR (400 MHz, d$_6$-DMSO) δ (ppm)**: 8.84 (d, J=7 Hz, 2H), 8.49 (d, J=8 Hz, 4H), 7.76 (t, J=7 Hz, 1H), 7.68 (t, J=8 Hz, 4H). **^{19}F NMR (376 MHz, d$_6$-DMSO) δ (ppm)**: -108.46. **Anal. Calcd. For $C_{21}H_{11}F_4N_3$ (%)**: C 66.14, H 2.91, N 11.02; **Found**: C 66.32, H 2.88, N 10.76.

Synthesis of *2,4,6-tris(3-(3,3'',6,6''-tetra-tert-butyl-9'H-[9,3':6',9''-tercarbazol]-9'-yl)phenyl)-1,3,5-triazine* (**tBuCz3mTRZ**).

A mixture of 3,3'',6,6''-tetrakis(*tert*-butyl-9'H-9,3':6',9''-tercarbazole) (**G2tBuCzH**) (1.19 g, 1.65 mmol, 3.3 equiv.), 2,4,6-tris(3-bromophenyl)-1,3,5-triazine (273 mg, 0.5 mmol, 1 equiv.), tetrakis(triphenylphosphine)palladium(0) (67 mg, 0.075 mmol, 0.15 equiv.), Xphos (72 mg, 0.15 mmol, 0.3 equiv.) and sodium *tert*-butoxide (432 mg, 4.5 mmol, 9 equiv.) in degassed anhydrous toluene (40 mL) was refluxed for 24 h. After cooling to room temperature, the mixture was filtered and washed with both brine and DCM (each 3×20 mL). The combined organic layers were dried with anhydrous sodium sulphate and concentrated under reduced pressure. The crude mixture was purified by silica gel flash column chromatography using hexane: DCM = 20: 1 as eluent to afford the desired compound as a green solid (950 mg). **Yield**: 77%. **R_f:** 0.4 (hexane: DCM = 2:1 on silica plate). **Mp**: no melting point was observed within the temperature range of 20 °C to 350 °C. **^{1}H NMR (400 MHz, Chloroform-*d*)**: δ 9.22 (t, J = 1.9 Hz, 3H), 9.00 (dt, J = 7.8, 1.4 Hz, 3H), 8.36 – 8.27 (m, 6H), 8.22 – 8.14 (m, 12H), 8.04 (ddd, J = 7.9, 2.2, 1.3 Hz, 3H), 7.97 (t, J = 7.8 Hz, 3H), 7.78 – 7.71 (m, 6H), 7.68 (dd, J = 8.7, 2.0 Hz, 6H), 7.47 (dd, J = 8.7, 1.9 Hz, 12H), 7.38 (dd, J = 8.5, 0.7 Hz, 12H), 1.48 (s, 108H). **^{13}C NMR (101 MHz, CDCl3)**: δ 171.62, 142.63, 140.10, 138.07, 131.76, 131.21, 130.94, 128.90, 127.91, 126.17, 124.14, 123.61, 123.17, 119.43, 116.28, 111.09, 109.07, 79.27, 76.91, 76.72, 34.75, 32.06. **MALDI-TOF-MS** (m/z): $[M]^+$ Calculated: 2468.40, Found: 2468.36. **Anal. Calcd. For $C_{177}H_{174}N_{12}$ (%)**: C, 86.09; H, 7.10; N, 6.81; **Found**: C, 86.01; H, 7.03; N, 6.81.

Synthesis of *9',9'''',9''''''',9''''''''''-((6-phenyl-1,3,5-triazine-2,4-diyl)bis(benzene-5,1,3-triyl))tetrakis(3,3'',6,6''-tetra-tert-butyl-9'H-9,3':6',9''-tercarbazole)* (**tBuCz4mTRZ**).

Under nitrogen, a mixture of 3,3'',6,6''-tetrakis(*tert*-butyl-9'H-9,3':6',9''-tercarbazole) (**G2tBuCzH**) (794 mg, 1.1 mmol, 4.4 equiv.), 2,4-bis(3,5-difluorophenyl)-6-phenyl-1,3,5-triazine (95 mg, 0.25 mmol, 1 equiv.) and cesium carbonate (652 mg, 2 mmol, 8 equiv.) in dry DMF (20 ml) was reflux for 24 h. After cooling to room temperature, the reaction was extracted with chloroform and washed with water (3 × 30 mL). The organic phase was dried over Na_2SO_4 and concentrated under reduced pressure. The crude product was purified by column chromatography on silica gel using 1:10 dichloromethane/hexane as eluent and then further purification was performed with preparative GPC column using THF as eluent to afford a yellow solid (360 mg). **Yield**: 45%. **R*f***: 0.35 (Hexane: DCM=2:1 on silica plate). **Mp**: no melting point was observed within the temperature range of 20 °C to 350 °C. **^{1}H NMR (400 MHz, Chloroform-*d*)**: δ 9.35 (d, *J* = 2.0 Hz, 4H), 8.93 – 8.87 (m, 2H), 8.44 (t, *J* = 2.0 Hz, 2H), 8.35 – 8.28 (m, 8H), 8.16 (dd, *J* = 1.9, 0.7 Hz, 16H), 7.96 – 7.87 (m, 8H), 7.72 (dd, *J* = 8.7, 2.0 Hz, 9H), 7.69 – 7.61 (m, 2H), 7.41 (dd, *J* = 8.7, 1.9 Hz, 16H), 7.34 (dd, *J* = 8.6, 0.6 Hz, 16H), 1.44 (s, 144H). **^{13}C NMR (101 MHz, $CDCl_3$)**: δ 142.71, 140.09, 139.97, 131.77, 126.37, 124.51, 123.62, 123.21, 119.58, 116.29, 111.00, 108.99, 77.35, 77.04, 76.72, 34.72, 32.02. **MALDI-TOF-MS** (m/z): $[M]^+$ Calculated: 3189.44, Found: 3189.43. **Anal. Calcd. For $C_{229}H_{227}N_{15}$ (%)**: C, 86.24; H, 7.17; N, 6.59; **Found**: C, 86.11; H, 7.04; N, 6.57.

Electrochemistry measurements

Cyclic Voltammetry (CV) analysis was performed on an Electrochemical Analyzer potentiostat model 620E from CH Instruments at a sweep rate of 100 mV/s. Differential pulse voltammetry (DPV) was conducted with an increment potential of 0.004 V and a pulse amplitude, width, and period of 50 mV, 0.05, and 0.5 s, respectively. Samples were prepared as dimethylformamide (DMF) solutions, which were degassed by sparging with DMF-saturated argon gas for 5 minutes prior to measurements. All measurements were performed using 0.1 M DMF solution of tetra-*n*-butylammonium hexafluorophosphate, [nBu$_4$N]PF$_6$]. An Ag/Ag$^+$ electrode was used as the reference electrode while a glassy carbon electrode and a platinum wire were used as the working electrode and counter electrode, respectively. The redox potentials are reported relative to a saturated calomel electrode (SCE) with a ferrocenium/ferrocene (Fc/Fc$^+$) redox couple as the internal standard (0.45 V vs SCE).[2]

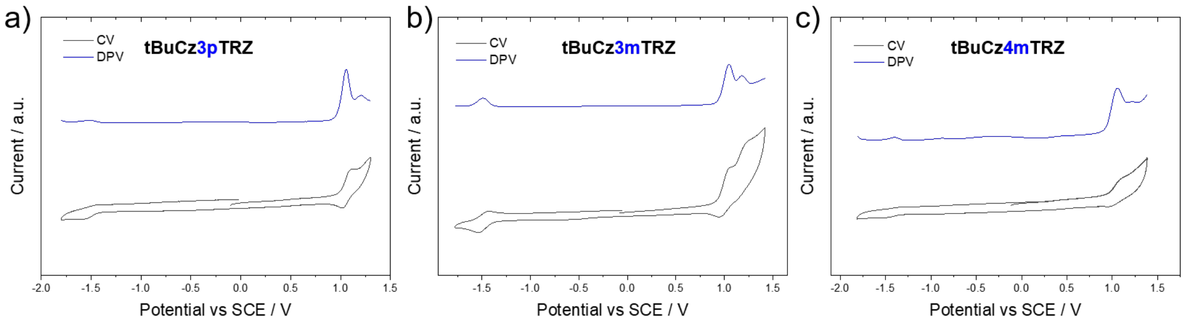

Figure S1. Cyclic voltammogram (CV) and differential pulse voltammetry (DPV) of **tBuCz3pTRZ**, **tBuCz3mTRZ** and **tBuCz4mTRZ** in degassed DMF with 0.1 M [nBu$_4$N]PF$_6$ as the supporting electrolyte and Fc/Fc$^+$ as the internal reference (0.45 V vs SCE).[2]

Table S1. Summary of literature-reported solution-processed non-doped OLED performance with TADF dendrimers as emitters.

Emitting layer	Peak [nm]	FWHM [nm]	V_{on} [a)] [V]	CE [cd A^{-1}]	PE [lm W^{-1}]	EQE_{max} [%]	EQE_{100} [%]	CIE (x,y)	Max. Luminance [cd m^{-2}]	Ref
**G2TAZ**	500/-	-/-	3.3/3.7	-/15.6	6/11.5	2.4/6.3	-/6.2	(0.251,0.493)/-	~1000/1243	[3]/[1a]
**G3TAZ**	515	-	3.5	-	8.5	3.4	-	(0.266,0.485)	~1000	[3]
**G4TAZ**	510	-	3.5	-	3.1	1.5	-	(0.232,0.368)	~200	[3]
**tBuG2TAZ**	500	-	3.5	25.4	16.1	9.5	9.4	-	2423	[1a]
**PhG2TAZ**	500	-	3.2	23.1	17.3	8.2	8.0	-	2316	[1a]
**MeG2TAZ**	500	-	3.0	26.5	21.5	9.4	9.4	-	2235	[1a]
**G2B**	500	-	3.4	14.0	11.5	5.7	-	(0.26, 0.48)	~1000	[4]
**G3B**	516	-	3.7	7.7	5.7	2.9	-	(0.31, 0.50)	~500	[4]
**CzDMAC-DPS**	502	-	3.6	30.6	24.0	12.2	6.9	(0.22, 0.44)	~2000	[5]
**DCzDMAC-DPS**	480	-	5.2	3.8	2.0	2.2	1.5	(0.18, 0.27)	~500	[5]
**CDE1**	552	114	4.4	38.9	17.3	13.8	-	(0.40, 0.54)	~10 000	[6]
**CDE2**	522	118	7.7	-	-	5.2	-	(0.32, 0.51)	2512	[6]
**TZ-Cz**	520	-	4.0	20.0	-	6.5	-	(0.24, 0.51)	18 200	[7]
**TZ-3Cz**	520	-	3.6	30.5	-	10.1	-	(0.24, 0.51)	22 950	[7]
**POCz-DPS**	480	-	5.4	12.6	-	7.3	-	(0.18, 0.30)	2700	[8]
**G-TCTA**	550	-	4.4	1.40	0.93	0.50	0.26	(0.46, 0.52)	1200	[9]
**G-mCP**	550	-	2.7	44.5	46.6	16.5	14.6	(0.42, 0.55)	18 800	[9]
**2CzSO**	516	96	4.7	-	-	10.7	-	(0.24, 0.49)	4706	[10]
**3CzSO**	510	90	4.3	-	-	6.3	-	(0.29, 0.52)	3531	[10]
**TB2CZ-ACTRZ**	520	-	4	30.8	24.2	9.5	-	(0.32, 0.57)	2336	[11]
**TB14CZ-ACTRZ**	496	-	4.5	20.7	14.5	8.1	-	(0.22, 0.43)	2770	[11]
**G2**	~430	-	4.8	4.1	1.6	-	-	(0.15, 0.12)	<1000	[12]
**G3**	~440	-	5.2	1.07	0.49	-	-	(0.19, 0.15)	<1000	[12]
**MPPA-Cz**	728	-	6.4	-	-	0.064	-	(0.70, 0.29)	24	[13]
**MPPA-3Cz**	715	-	6.2	-	-	0.254	-	(0.69, 0.30)	135	[13]

Table S2. Electrochemical data and theoretical properties of **tBuCz3pTRZ**, **tBuCz3mTRZ** and **tBuCz4mTRZ**

	Electrochemical potential [a]			Theoretical [e]					
	E_{ox} [b] / V	E_{red} [c] / V	$\Delta E_{H\text{-}L}$ [d] / V	HOMO / eV	LUMO / eV	f [f]	S_1 / eV	T_1 / eV	ΔE_{ST} / eV
**tBuCz3pTRZ**	1.06	-1.51	2.57	-5.32	-2.34	0.2533	2.60	2.53	0.07
**tBuCz3mTRZ**	1.05	-1.49	2.54	-5.29	-2.37	0.0125	2.51	2.46	0.05
**tBuCz4mTRZ**	1.06	-1.40	2.46	-5.29	-2.52	0.0055	2.38	2.35	0.03

[a] Potential values were obtained from the DPV peak values and referenced with respect to SCE (Fc/Fc^+ = 0.45 eV), the measurement was performed in DMF; [b] Oxidation potential calculated from the DPV peak value; [c] Reduction potential calculated from the DPV peak value; [d] $\Delta E_{H\text{-}L} = E_{ox}\text{-}E_{red}$; [e] Calculated in the gas phase at PBE0/6-31G(d, p) level; [f] Calculated oscillator strength of S_1.

The electrochemical properties of **tBuCz3pTRZ**, **tBuCz3mTRZ** and **tBuCz4mTRZ** were investigated by cyclic voltammetry (CV) and differential pulse voltammetry (DPV) in dimethylformamide (DMF). As shown in **Figure S1**, all dendrimers show two similar irreversible oxidation waves (1.06, 1.21 V for **tBuCz3pTRZ**; 1.05, 1.18 V for **tBuCz3mTRZ**; 1.06, 1.21 V for **tBuCz4mTRZ** according to DPV peak values), which can be ascribed to the hole delocalization over the inner and peripheral carbazoles of the tercarbzole donor dendron unit. The calculated HOMO levels for **tBuCz3pTRZ**, **tBuCz3mTRZ**, and **tBuCz4mTRZ** are -5.41 eV, -5.40 eV and -5.41 eV, respectively, which are very close to the simulated HOMO levels (**Table S2**). The reduction was found to be irreversible as well for all the dendrimers and the LUMOs for **tBuCz3pTRZ**, **tBuCz3mTRZ**, and **tBuCz4mTRZ** were calculated to be -2.84 eV, -2.86 eV, and -2.95 eV respectively, according to the peak value of the DPV.

OLED fabrication and characterization

The OLED devices were fabricated using a bottom-emitting architecture. A pre-patterned indium tin oxide (ITO) glass substrate with a sheet resistance of 15 Ω square^{-1} was pre-cleaned carefully with detergent and deionized water and then exposed to UV-ozone for 15 min. PEDOT:PSS was spin-coated onto the clean ITO substrate as the hole-injection layer, followed by thermal treatment under 120 °C for 30 min. Then a 10 mg/mL chlorobenzene solution of our dendrimers was spin-coated to form a 35-45 nm thick emissive layer (EML) and annealed at 120 °C for 10 min to remove residual solvent before transfer to the vacuum chamber. A 40 nm-thick electron-transporting layer (ETL) of Tm3PyPB was then vacuum deposited at a rate of 1 Å/s, which was controlled *in situ* using quartz crystal monitors. The electron injection layer LiF was deposited at a rate of 0.1 Å/s while the Al cathode was deposited at a rate of 10 Å/s through the shadow mask defining the top electrode. The spatial overlap of the anode and cathode electrodes determined the active area of the OLED, which was estimated to be 4 mm^2. Electroluminescence (EL), CIE color coordinates, and spectra were obtained via a Spectrascan PR655 photometer, and the luminance-current-voltage characteristics were determined with a computer-controlled Keithley 2400 Source meter. EQE was calculated from the current density, luminance, and EL spectrum, assuming Lambertian emission distribution.

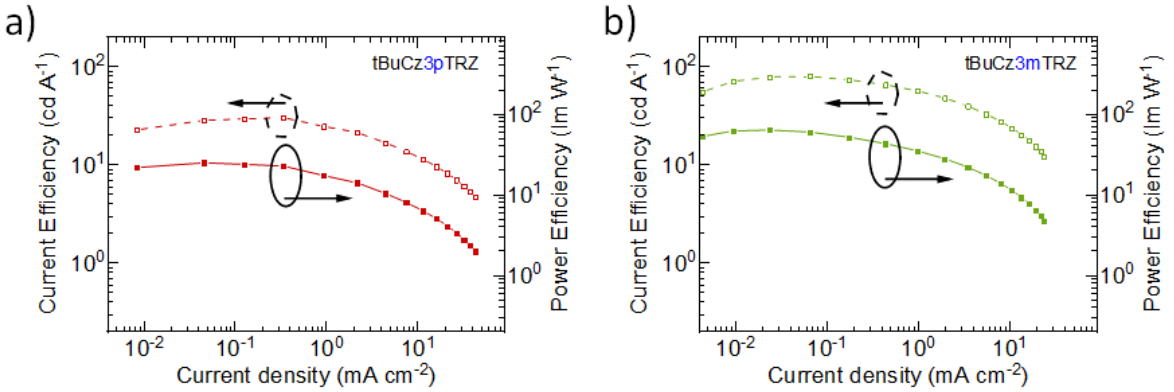

Figure S2. Current and power efficiency versus current density curves for **tBuCz3pTRZ** and **tBuCz3pTRZ**.

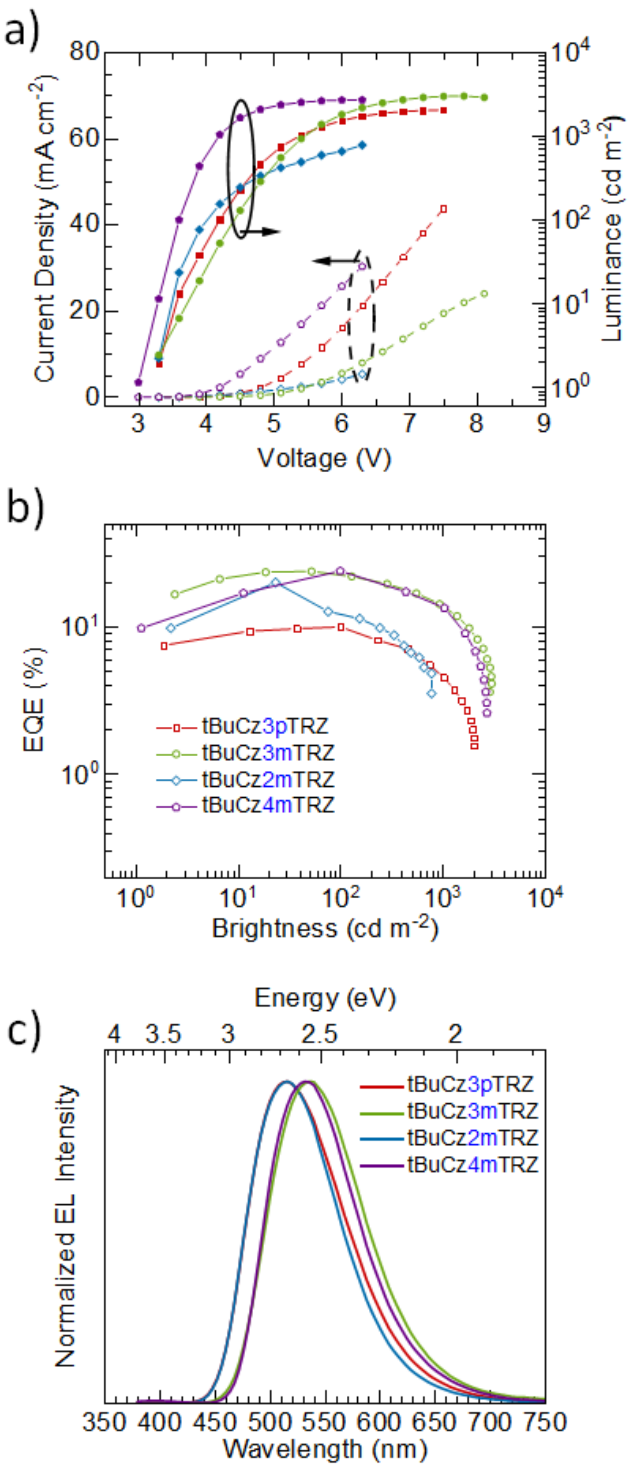

Figure S3. a) Current density-voltage-luminance (J-V-L) curves, b) EQE vs brightness curve and c) electroluminescence (EL) spectra for **tBuCz3pTRZ**, **tBuCz3mTRZ**, **tBuCz2mTRZ** and **tBuCz4mTRZ**.

Table S3. Summary of device performance

Emitting layer a)	λ_{EL} b) / nm	FWHM c) / nm	V_{on} d) / V	CE e) / cd A^{-1}	PE f) / lm W^{-1}	EQE_{max} g) / %	EQE_{100} h) / %	EQE_{500} / %	CIE i) / (x,y)	L_{max} j) / cd m^{-2}
**tBuCz3pTRZ**	516	100	3.3	30.0	24.5	10.0	10.0	6.9	0.27, 0.52	2039
**tBuCz2mTRZ**	516	95	3.3	59.9	52.2	19.9	12.0	6.4	0.27, 0.53	777
**tBuCz3mTRZ**	536	100	3.3	78.7	61.0	23.7	22.2	17.5	0.35, 0.57	2994
**tBuCz4mTRZ**	536	93	3.0	81.5	71.1	23.8	23.8	17.0	0.36, 0.58	2691

a) The device structure is: ITO/PEDOT:PSS (35 nm)/ dendrimer (40 nm)/TmPyPB (40 nm)/LiF (1 nm)/ Al (100 nm); b) Emission maximum; c) Full width at half maximum, FWHM; d) turn-on voltage at the luminance of 1 cd m^{-2}; e) Maximum current efficiency; f) Maximum power efficiency; g) Maximum external quantum efficiency; h) At the luminance of 100 cd m^{-2}; i) Commission Internationale de l'Éclairage; j) Maximum luminance.

Photophysical characterization

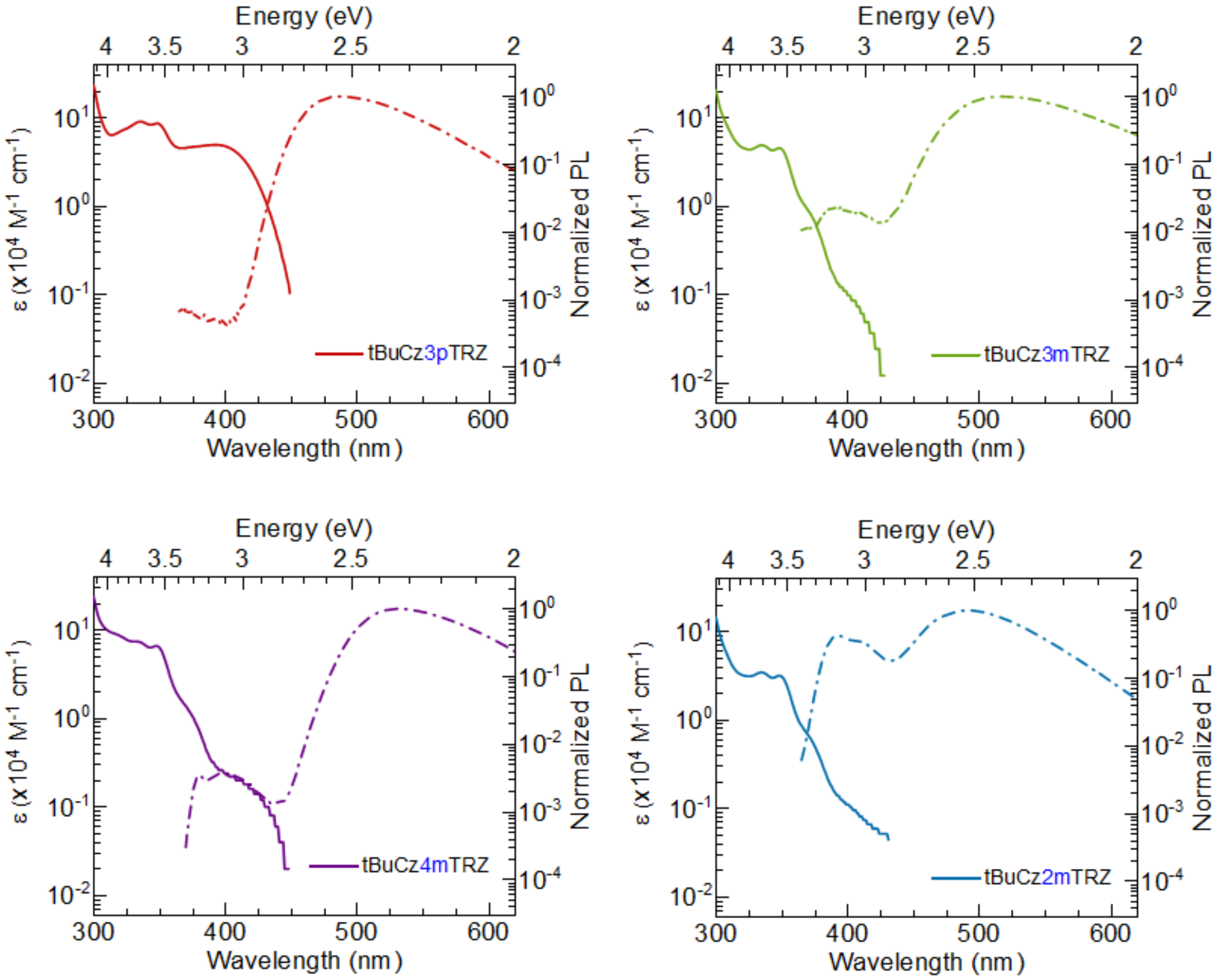

Figure S4. RT molar absorption and emission (λ_{exc} = 300 nm) for the dendrimers under investigation in toluene solution (concentration = 0.05 mg/mL).

Table S4. Summary of PL maxima in solvatochromic study of **tBuCz3pTRZ**, **tBuCz2mTRZ**, **tBuCz3mTRZ** and **tBuCz4mTRZ**

Solvent	λ_{PL} [a] / nm			
	**tBuCz3pTRZ**	**tBuCz2mTRZ**	**tBuCz3mTRZ**	**tBuCz4mTRZ**
Hexane (Hex)	442	439	483	496
Toluene (Tol)	486	492	517	532
Diethyl ether (Et_2O)	517	509	543	541
Ethyl acetate (EA)	558	546	584	569
CH_2Cl_2	590	574	619	606

[a] Peak value of PL spectra obtained under aerated conditions at 300 K, concentration of 3×10^{-5} M, λ_{exc} = 300 nm.

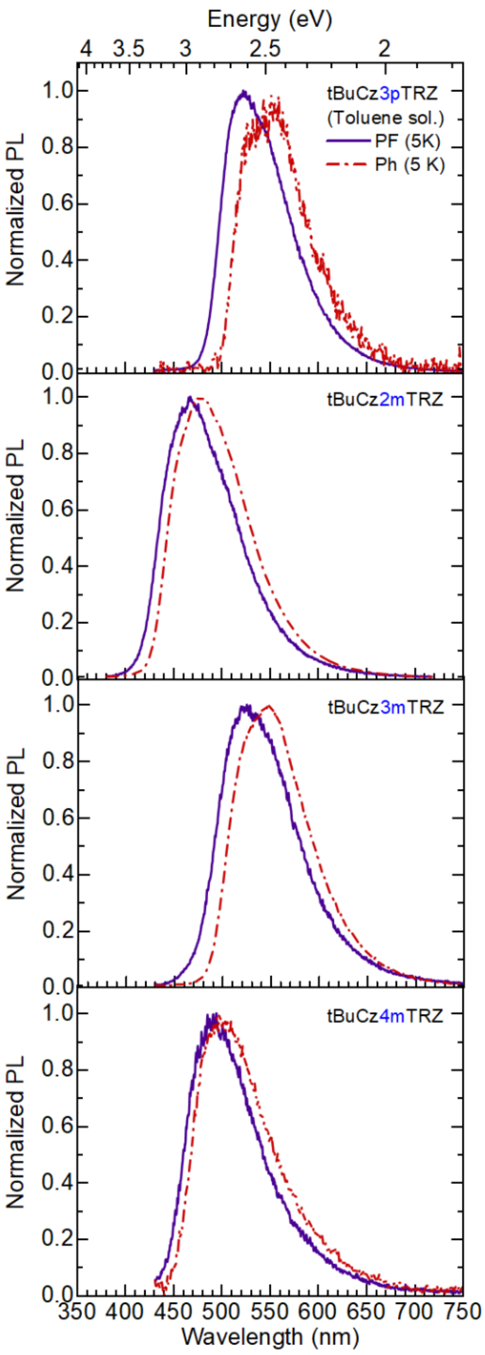

Figure S5. Prompt fluorescence (delay time: 10 ns, gating time: 7 ns) and phosphorescence (delay time: 10 ms, gating time: 1 ms) acquired in toluene glass at 5 K (λ_{exc} = 355 nm). The singlet (triplet) energies at 5 K are determined to be 2.57 eV (2.47 eV), 2.62 eV (2.54 eV), 2.94 eV (2.88 eV) and 2.81 eV (2.76 eV) and, the ΔE_{ST} values are estimated to be 100 meV, 80 meV, 60 meV and 50 meV for **tBuCz3pTRZ**, **tBuCz3mTRZ**, **tBuCz2mTRZ** and **tBuCz4mTRZ**, respectively.

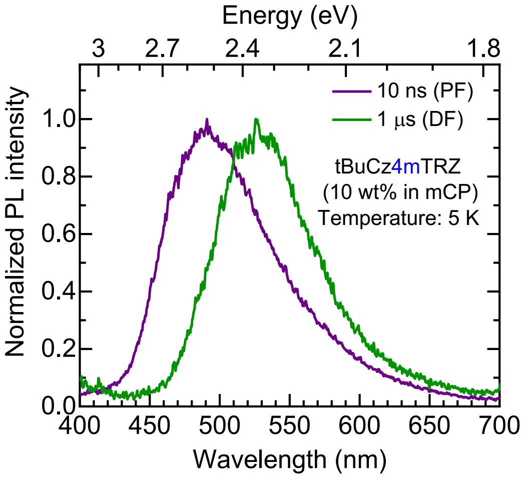

Figure S6. Comparison of prompt fluorescence (PF) and delayed fluorescence (DF) spectra for 10 wt% doped dendrimer films in mCP.

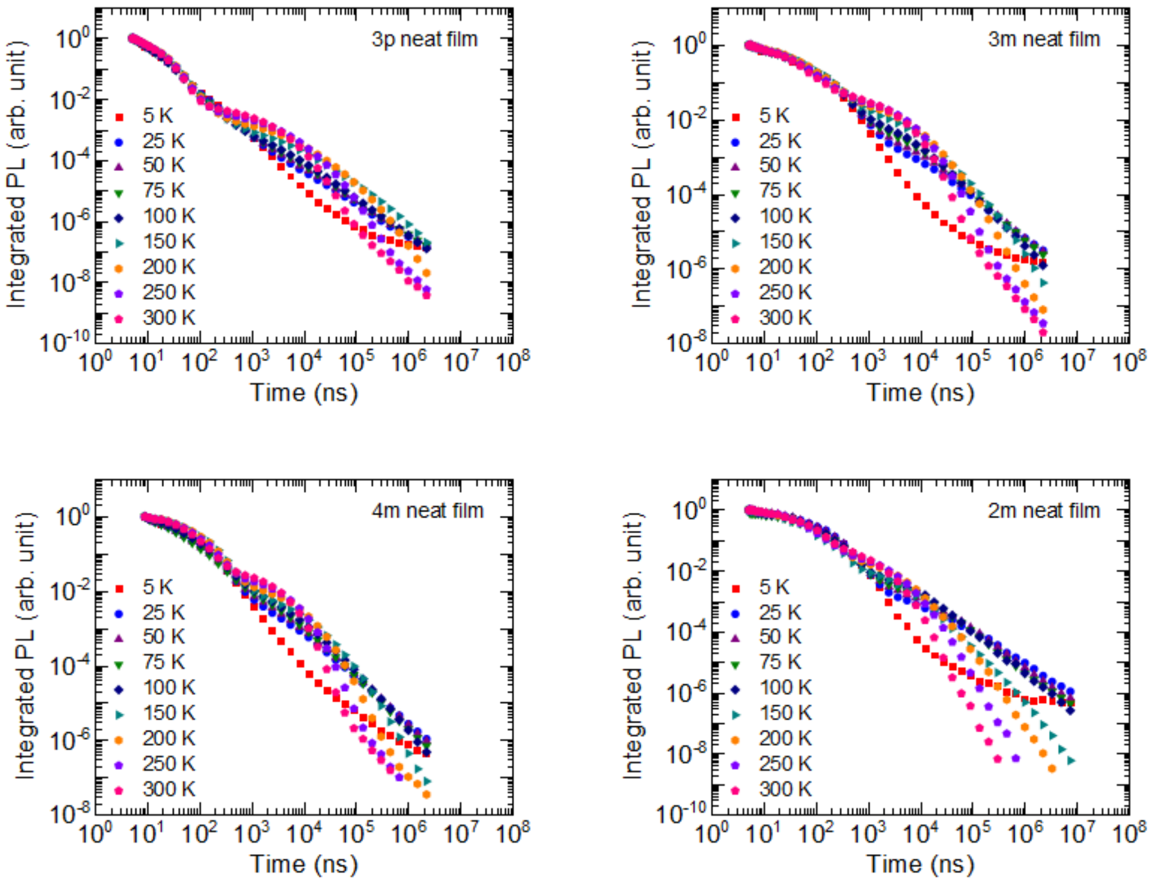

Figure S7. Temperature dependent PL decays of dendrimer neat films.

Determination of photophysical rate constants

- The rate constants were determined according to the method described in literature. [14]

- Prompt lifetime (τ_{PF}) and delayed lifetime (τ_{DF}) were determined from the monoexponential fits of the prompt and delayed components of the PL decay at RT. Photoluminescence quantum efficiency (ϕ_{PL}) was determined under N_2 atmosphere. ϕ_{DF}/ϕ_{PF} was determined from the ratio of the corresponding integrals in the PL decay curves.

- Since, $\phi_{PL} = \phi_{PF} + \phi_{DF} \Rightarrow \phi_{PF} = \frac{\phi_{PL}}{1+\phi_{DF}/\phi_{PF}}$ and $\phi_{DF} = \phi_{PL} - \phi_{PF}$.

- Assuming negligible nonradiative singlet decay rate, ISC quantum efficiency, $\phi_{ISC} = 1 - \phi_{PF}$.

- RISC quantum efficiency is then determined as: $\phi_{rISC} = \frac{\phi_{DF}}{\phi_{ISC}\,\phi_{PL}}$

- Radiative singlet decay rate, $k_F = \frac{\phi_{PF}}{\tau_{PF}}$.

- Intersystem crossing rate, $\mathrm{k_{ISC}} = \frac{\phi_{ISC}}{\tau_{PF}}$.

- Reverse intersystem crossing rate, $\mathrm{k_{RISC}} = \frac{1}{\phi_{ISC}\,\tau_{DF}}\left(\frac{\phi_{DF}}{\phi_{PF}}\right)$.

- Nonradiative triplet decay rate, $\mathrm{k_{NR}^{T}} = \frac{\mathrm{k_{rISC}}}{\phi_{rISC}} - \mathrm{k_{rISC}}$.

Theoretical calculations

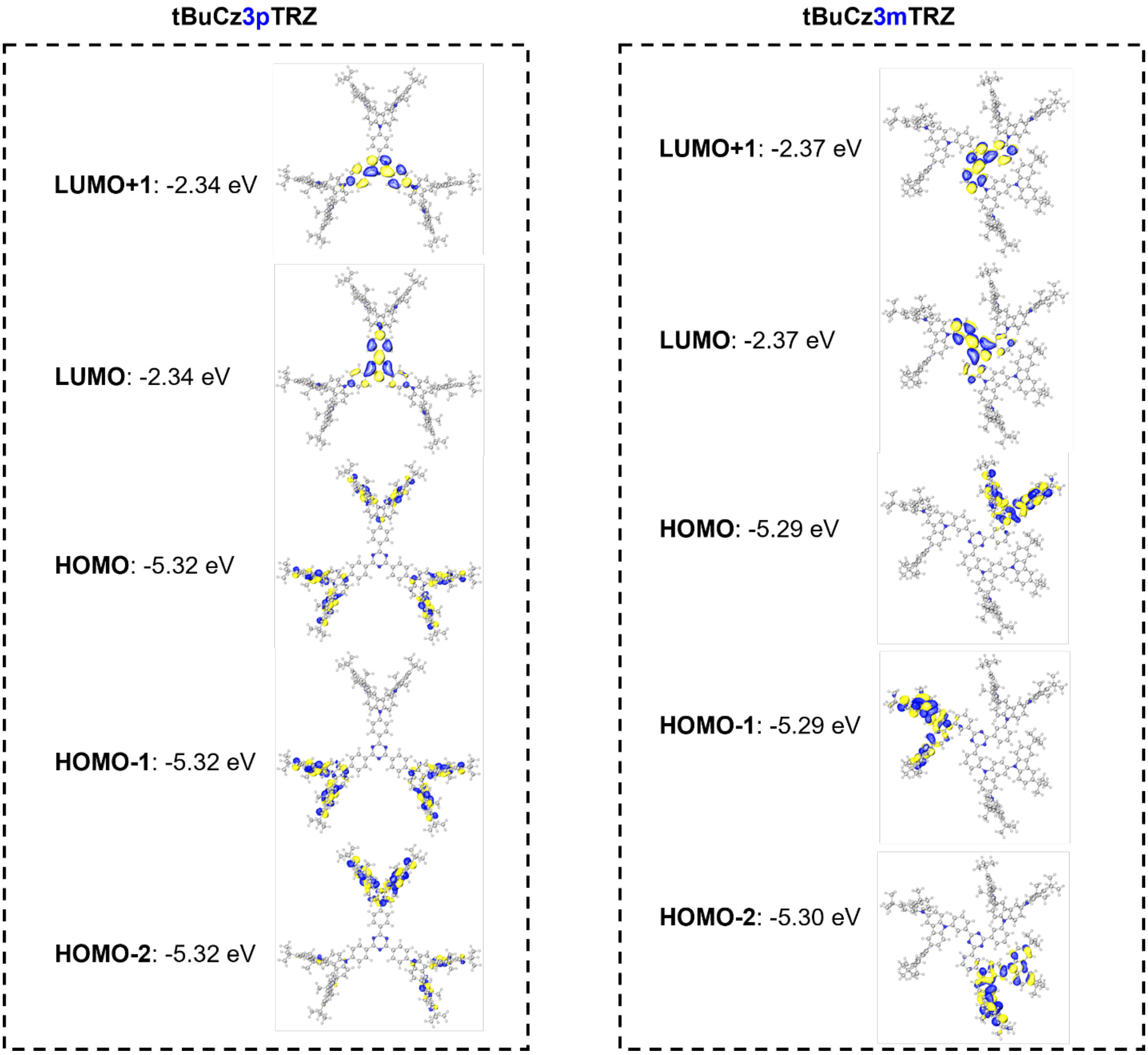

Figure S8. Calculated distribution of molecular orbitals for **tBuCz3pTRZ** and **tBuCz3mTRZ**. (isovalue= 0.02)

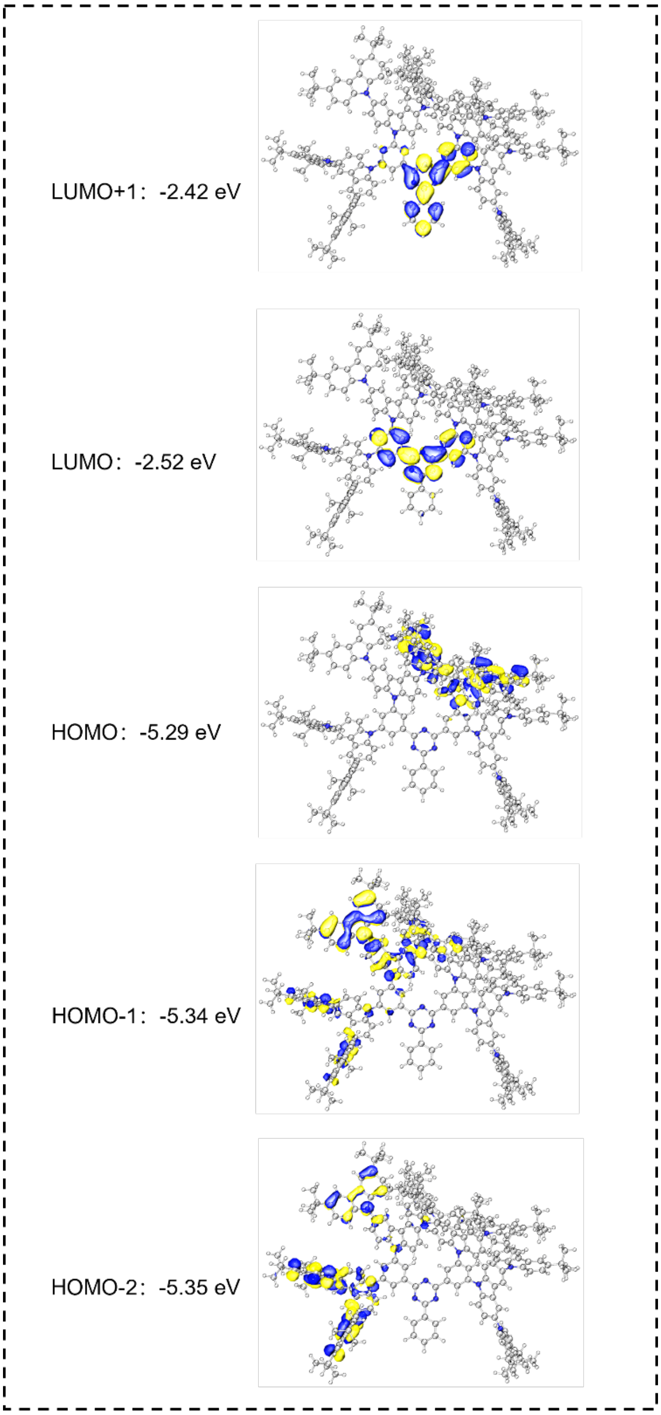

Figure S9. Calculated distribution of molecular orbitals for **tBuCz4mTRZ**. (isovalue= 0.02)

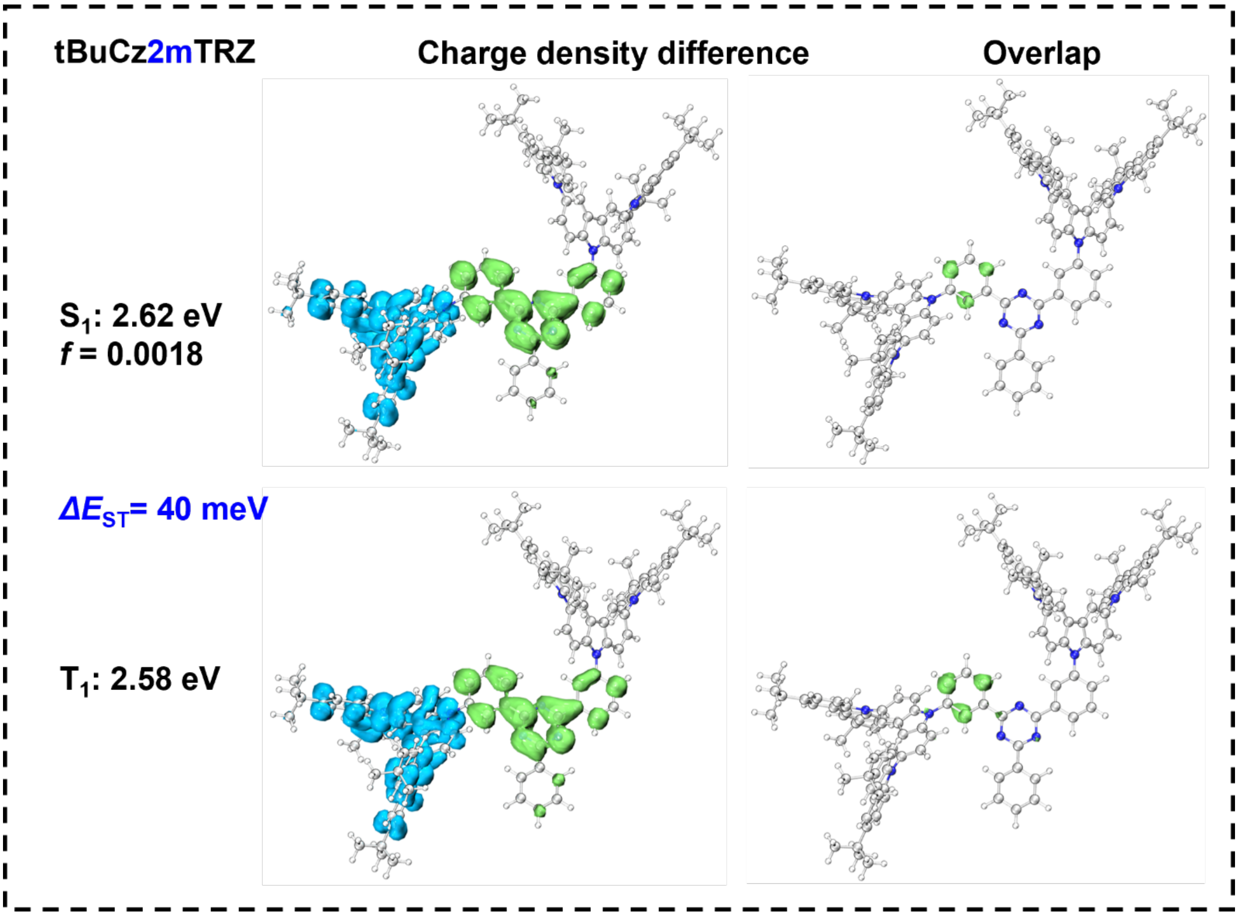

Figure S10. Pictorial representation of charge density difference and overlap of hole and electron for **tBuCz2mTRZ**. *f* indicates the oscillator strength and S_1/ T_1 are the calculated first singlet/ triplet energy. ΔE_{ST} refers to the energy difference between S_1 and T_1. (isovalue= 0.02)

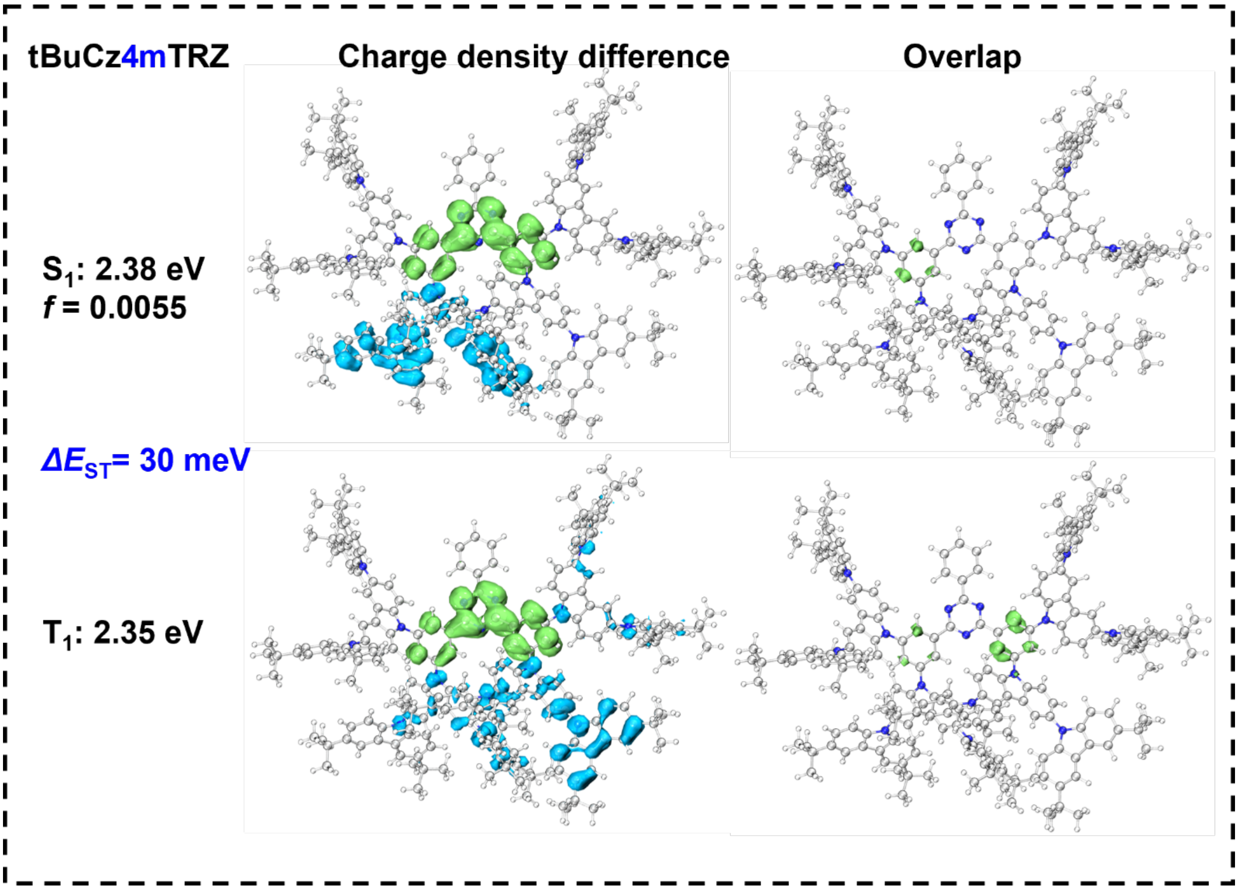

Figure S11. Pictorial representation of charge density difference and overlap of hole and electron for **tBuCz4mTRZ**. *f* indicates the oscillator strength and S_1/ T_1 are the calculated first singlet/ triplet energy. ΔE_{ST} refers to the energy difference between S_1 and T_1. (isovalue= 0.02)

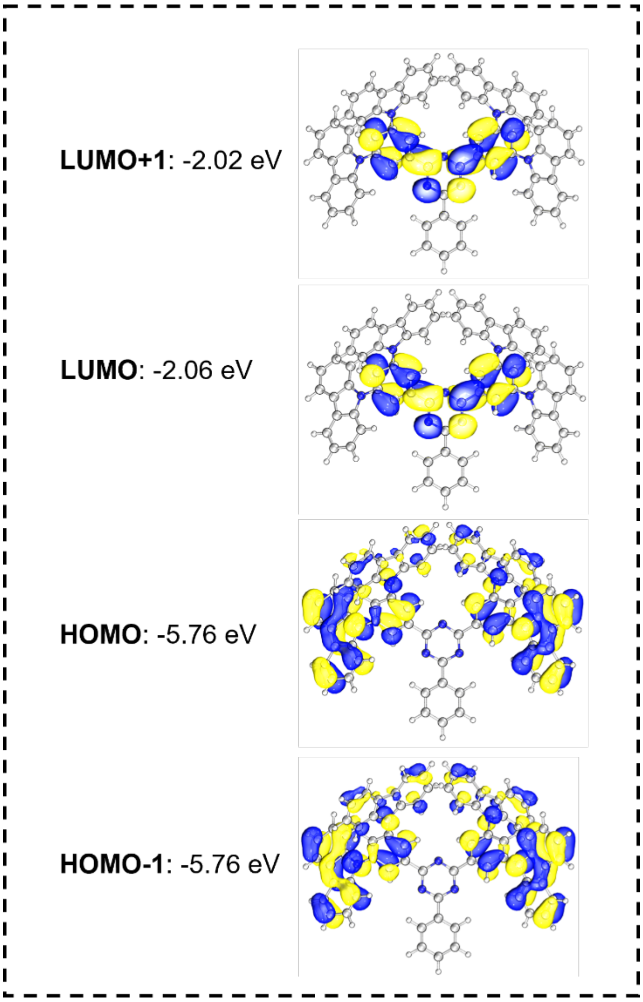

Figure S12. Calculated distribution of molecular orbitals for **4m-core**. (isovalue= 0.02)

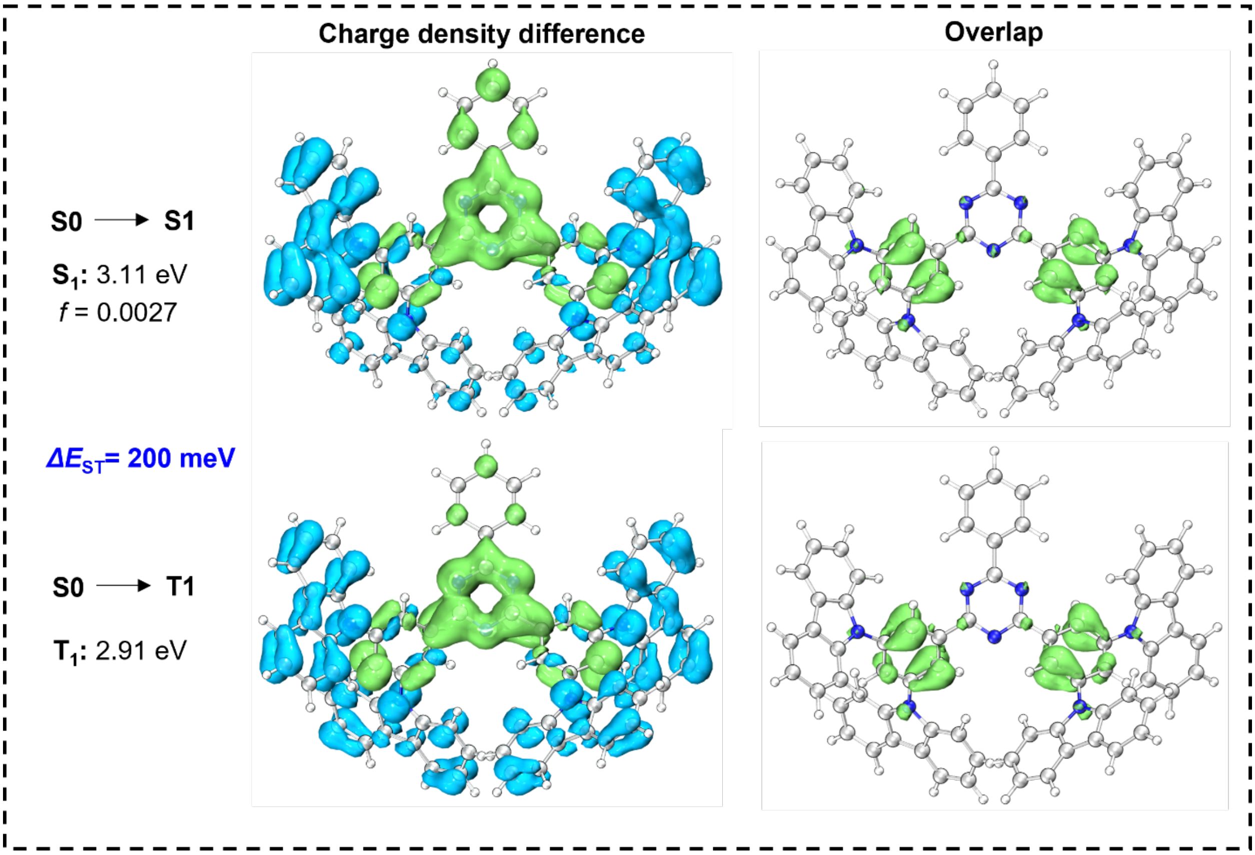

Figure S13. Pictorial representation of charge density difference and overlap of hole and electron for **4m-core**. *f* indicates the oscillator strength and S_1/ T_1 are the calculated first singlet/ triplet energy. ΔE_{ST} refers to the energy difference between S_1 and T_1. (isovalue= 0.02)

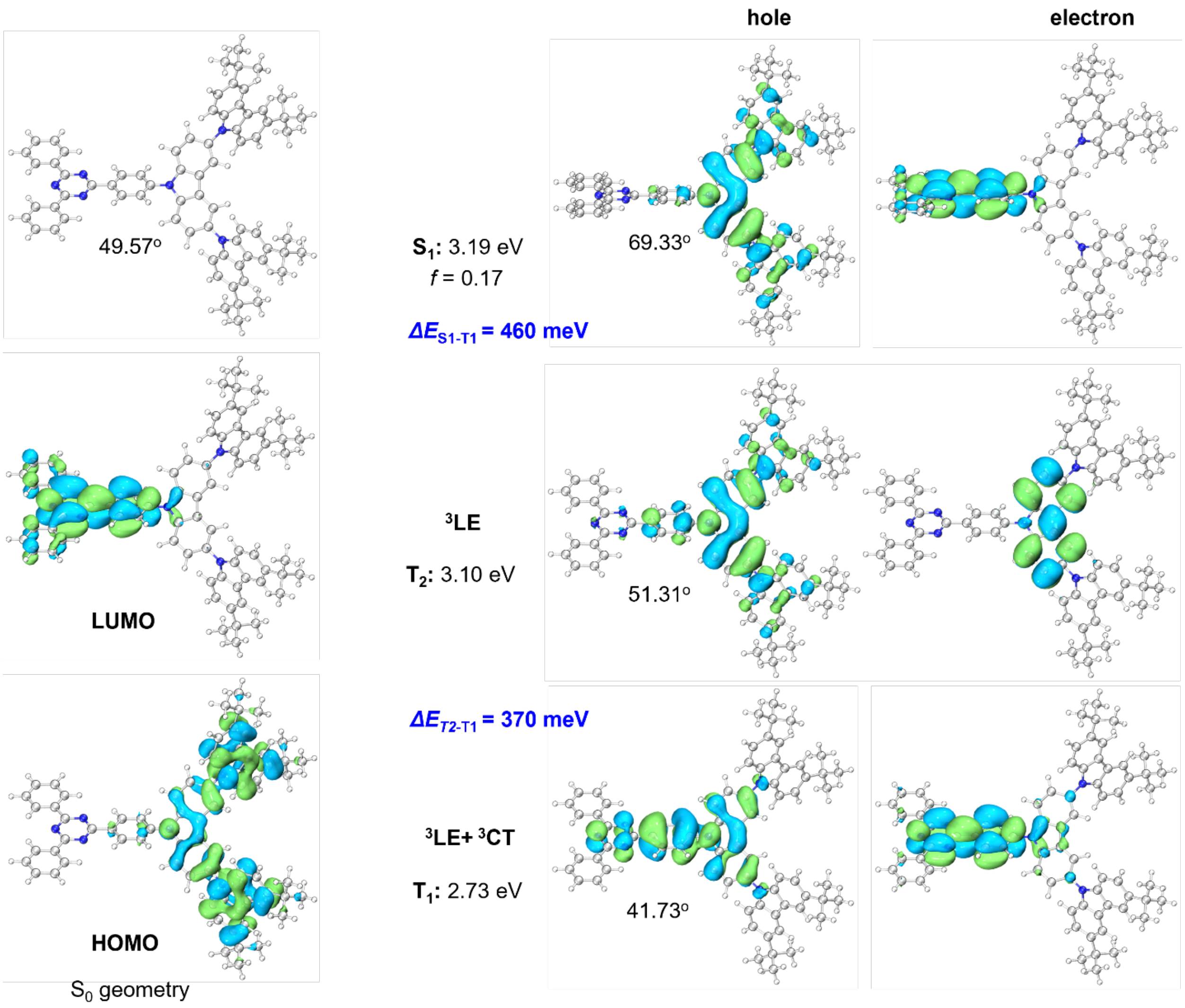

Figure S14. Natural transition orbital (NTO) pairs for the S1 and T1 states of **tBuCz1pTRZ**.

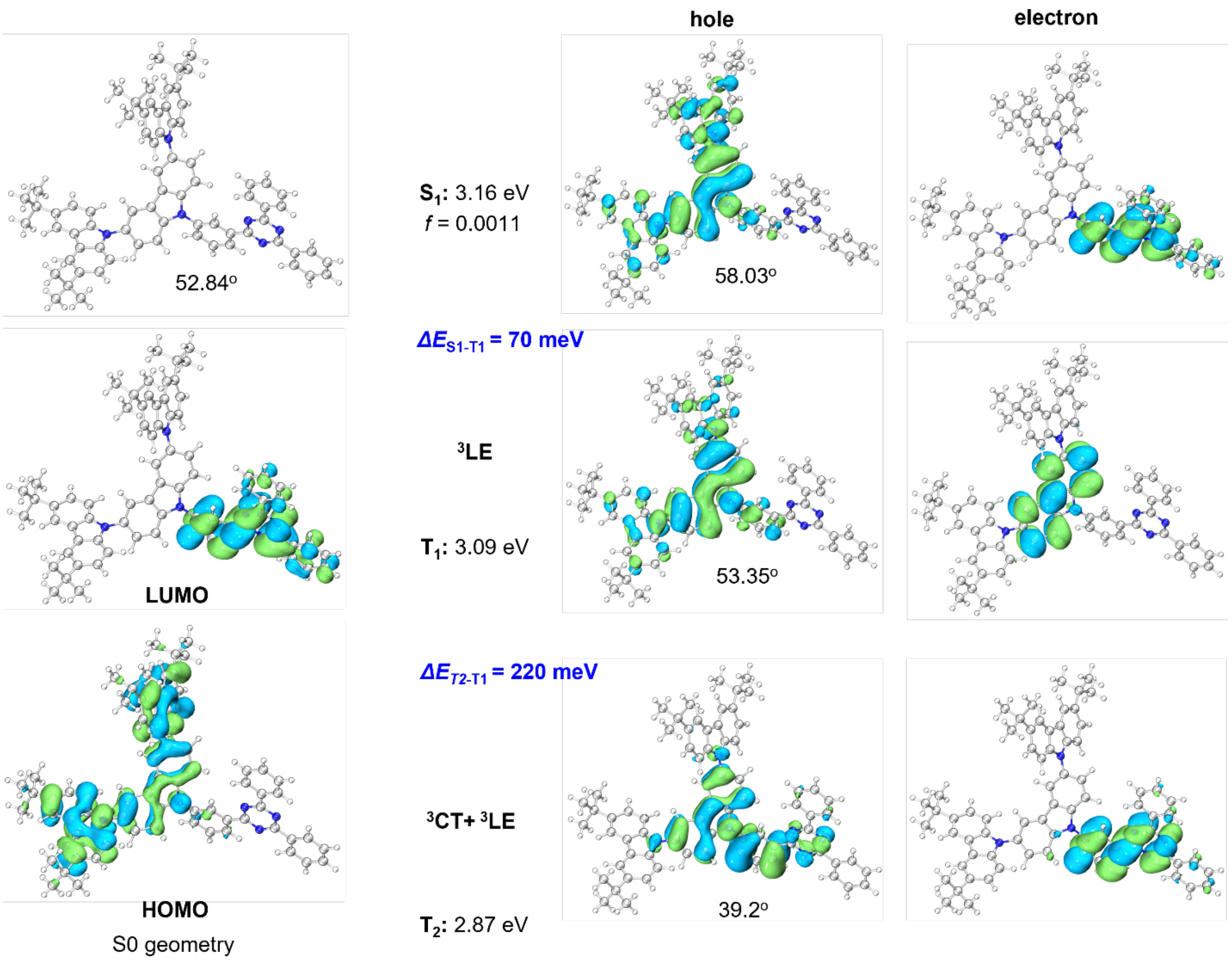

Figure S15. Natural transition orbital (NTO) pairs for the S1 and T1 states of **tBuCz1mTRZ**.

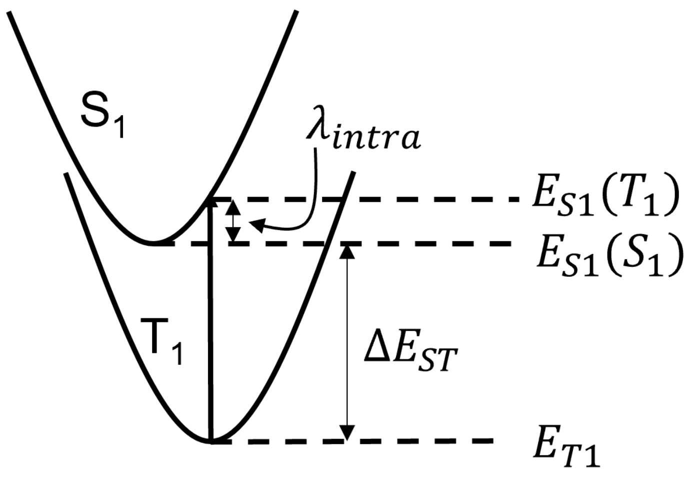

Figure S16. Marcus' parabolic free energy curves for the description of the reorganization energy.

Table S5. Reverse intersystem crossing rates determined from experiment and computation.

Parameter	tBuCz3pTRZ	tBuCz3mTRZ	Method	λ_{inter} (meV)
E_{ST} (meV)	100	80	Experiment	---
SOCME (cm^{-1})	0.31	0.53	Quantum chemical analysis	---
λ_{intra} (meV)	275	155		
k_{RISC} (s^{-1})	5×10^{5}	9×10^{6}	Eq. 1 and 2	0
k_{RISC} (s^{-1})	5×10^{4}	4×10^{5}	Experiment	---
k_{RISC} (s^{-1})	2.1×10^{4}	3.6×10^{5}	Eq. 1 and 2	300

Table S6. Selected low energy vertical transitions with corresponding oscillator strengths (*f*) and character assignments for compound **4m-core** as calculated at the TDA PBE0/6-31G(d, p) theory level.

State	E [eV]	*f*	Main contribution
T_1	2.91	0	H-1→LUMO (26%), HOMO→L+1 (58%)
T_2	2.91	0	H-1→L+1 (59%), HOMO→LUMO (26%)
T_3	3.02	0	H-3→LUMO (21%), H-2→L+1 (55%)
T_4	3.04	0	H-3→L+1 (56%), H-2→LUMO (23%)
S_1	3.11	0.0272	H-1→LUMO (40%), HOMO→L+1 (57%)
S_2	3.11	0.0192	H-1→L+1 (56%), HOMO→LUMO (41%)
T_5	3.20	0	H-1→LUMO (65%), HOMO→L+1 (30%)
T_6	3.20	0	H-1→L+1 (31%), HOMO→LUMO (65%)
S_3	3.21	0.0009	H-2→L+1 (20%), H-1→L+1 (28%), HOMO→LUMO (48%)
S_4	3.21	0.002	H-3→L+1 (15%), H-1→LUMO (52%), HOMO→L+1 (31%)
S_5	3.22	0.0134	H-3→L+1 (45%), H-2→LUMO (39%)
S_6	3.22	0.0016	H-3→LUMO (38%), H-2→L+1 (40%), H-1→L+1 (12%)
T_7	3.31	0	H-3→LUMO (70%), H-2→L+1 (26%)
T_8	3.31	0	H-3→L+1 (27%), H-2→LUMO (69%)
S_7	3.31	0.0012	H-3→L+1 (38%), H-2→LUMO (59%)
S_8	3.32	0.0002	H-3→LUMO (58%), H-2→L+1 (38%)
T_9	3.39	0	H-10→LUMO (74%)
T_{10}	3.44	0	H-7→L+4 (22%), H-6→L+2 (39%)
S_9	3.58	0	H-5→LUMO (41%), H-5→L+1 (35%), H-4→L+1 (15%)
S_{10}	3.58	0	H-5→L+1 (15%), H-4→LUMO (41%), H-4→L+1 (36%)

Table S7. Selected low energy vertical transitions with corresponding oscillator strengths (*f*) and character assignments for compound **tBuCz3pTRZ** as calculated at the TDA PBE0/6-31G(d, p) theory level.

State	E [eV]	*f*	Main contribution
T_1	2.53	0	H-2→LUMO (42%), H-1→L+1 (42%)
T_2	2.54	0	H-2→LUMO (18%), H-1→L+1 (17%), HOMO→LUMO (28%), HOMO→L+1 (15%)
T_3	2.54	0	H-2→L+1 (14%), H-1→LUMO (16%), HOMO→LUMO (14%), HOMO→L+1 (33%)
S_1	2.60	0.2533	H-2→LUMO (23%), H-1→L+1 (19%), HOMO→LUMO (42%)
S_2	2.60	0.2551	H-2→L+1 (17%), H-1→LUMO (21%), HOMO→L+1 (46%)
S_3	2.65	0	H-2→LUMO (48%), H-1→L+1 (47%)
T_4	2.69	0	H-2→LUMO (21%), H-1→L+1 (25%), HOMO→LUMO (52%)
T_5	2.69	0	H-2→L+1 (17%), H-1→LUMO (36%), HOMO→L+1 (45%)
T_6	2.69	0	H-2→L+1 (59%), H-1→LUMO (36%)
S_4	2.69	0	H-2→L+1 (39%), H-1→LUMO (55%)
S_5	2.69	0	H-2→LUMO (22%), H-1→L+1 (26%), HOMO→LUMO (50%)
S_6	2.69	0	H-2→L+1 (38%), H-1→LUMO (18%), HOMO→L+1 (43%)
T_7	2.75	0	H-4→LUMO (98%)
T_8	2.75	0	H-3→LUMO (32%), H-3→L+1 (66%)
T_9	2.75	0	H-5→LUMO (19%), H-5→L+1 (80%)
S_7	2.76	0.0061	H-4→LUMO (99%)
S_8	2.76	0.006	H-3→LUMO (32%), H-3→L+1 (67%)
S_9	2.76	0.0061	H-5→LUMO (19%), H-5→L+1 (80%)
T_{10}	2.83	0	H-3→LUMO (67%), H-3→L+1 (32%)
S_{10}	2.83	0	H-3→LUMO (67%), H-3→L+1 (33%)

Table S8. Selected low energy vertical transitions with corresponding oscillator strengths (*f*) and character assignments for compound **tBuCz3mTRZ** as calculated at the TDA PBE0/6-31G(d, p) theory level.

State	E [eV]	*f*	Main contribution
T_1	2.46	0	H-2→LUMO (11%), H-2→L+1 (76%)
T_2	2.47	0	HOMO→LUMO (31%), HOMO→L+1 (58%)
T_3	2.48	0	H-1→LUMO (88%)
S_1	2.51	0.0125	H-2→L+1 (86%)
S_2	2.52	0.0044	HOMO→LUMO (36%), HOMO→L+1 (55%)
S_3	2.52	0.0052	H-1→LUMO (89%)
T_4	2.56	0	H-2→LUMO (79%), H-2→L+1 (10%)
S_4	2.56	0.0013	H-2→LUMO (86%)
T_5	2.57	0	HOMO→LUMO (61%), HOMO→L+1 (33%)
S_5	2.58	0.0035	HOMO→LUMO (56%), HOMO→L+1 (39%)
T_6	2.58	0	H-1→L+1 (93%)
S_6	2.58	0.0034	H-1→L+1 (92%)
T_7	2.69	0	H-4→LUMO (73%), H-3→LUMO (20%)
S_7	2.69	0.0008	H-4→LUMO (72%), H-3→LUMO (21%)
T_8	2.69	0	H-4→L+1 (11%), H-3→LUMO (35%), H-3→L+1 (42%)
S_8	2.69	0.001	H-4→LUMO (10%), H-4→L+1 (11%), H-3→LUMO (33%), H-3→L+1 (42%)
T_9	2.70	0	H-5→L+1 (87%)
S_9	2.70	0.0009	H-5→L+1 (89%)
T_{10}	2.74	0	H-4→LUMO (10%), H-4→L+1 (12%), H-3→LUMO (41%), H-3→L+1 (34%)
T_{11}	2.74	0	H-4→L+1 (70%), H-3→L+1 (21%)

Table S9. Selected low energy vertical transitions with corresponding oscillator strengths (*f*) and character assignments for compound **tBuCz4mTRZ** as calculated at the TDA PBE0/6-31G(d, p) theory level.

State	E [eV]	*f*	Main contribution
**T_1**	2.35	0	H-1→LUMO (57%), HOMO→LUMO (32%)
**T_2**	2.37	0	H-1→LUMO (31%), HOMO→LUMO (56%)
**S_1**	2.38	0.0055	HOMO→LUMO (93%)
**S_2**	2.41	0.0125	H-1→LUMO (93%)
**T_3**	2.42	0	H-2→LUMO (90%)
**S_3**	2.45	0.0001	H-2→LUMO (94%)
**T_4**	2.45	0	H-3→LUMO (72%), H-3→L+1 (20%)
**S_4**	2.48	0.0041	H-3→LUMO (85%), H-3→L+1 (11%)
**T_5**	2.49	0	HOMO→L+1 (83%)
**S_5**	2.50	0.0016	HOMO→L+1 (92%)
**T_6**	2.56	0	H-4→LUMO (84%)
**S_6**	2.56	0.0003	H-4→LUMO (88%)
**T_7**	2.56	0	H-3→L+1 (13%), H-1→L+1 (70%)
**T_8**	2.57	0	H-3→LUMO (17%), H-3→L+1 (59%), H-1→L+1 (16%)
**S_7**	2.57	0.0052	H-1→L+1 (87%)
**S_8**	2.58	0.0056	H-3→LUMO (11%), H-3→L+1 (83%)
**T_9**	2.58	0	H-5→LUMO (95%)
**S_9**	2.58	0.0005	H-5→LUMO (94%)
**T_{10}**	2.59	0	H-7→LUMO (52%), H-2→L+1 (41%)
**S_{10}**	2.60	0.0043	H-7→LUMO (51%), H-2→L+1 (41%)
**T_{11}**	2.60	0	H-7→LUMO (40%), H-2→L+1 (49%)
**S_{11}**	2.60	0.0007	H-7→LUMO (40%), H-2→L+1 (49%)
**T_{12}**	2.62	0	H-6→LUMO (90%)
**S_{12}**	2.62	0.0003	H-6→LUMO (90%)
**T_{13}**	2.67	0	H-4→L+1 (94%)
**S_{13}**	2.67	0.0004	H-4→L+1 (94%)
**T_{14}**	2.71	0	H-6→L+1 (88%)
**S_{14}**	2.71	0.0008	H-6→L+1 (88%)
**T_{15}**	2.73	0	H-5→L+1 (93%)
**S_{15}**	2.73	0.0005	H-5→L+1 (92%)

NMR spectra

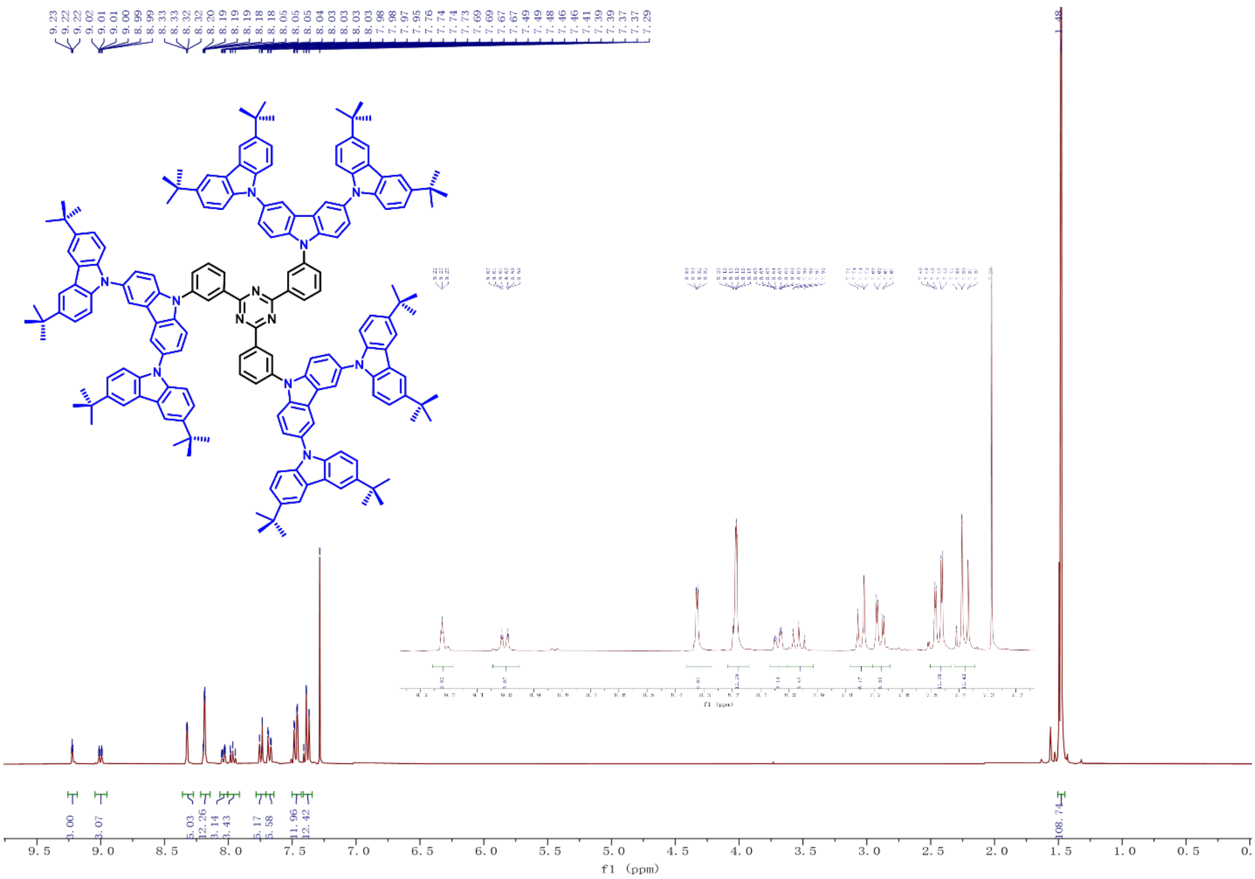

Figure S17. ^{1}H NMR spectrum of **tBuCz3mTRZ** in $CDCl_3$.

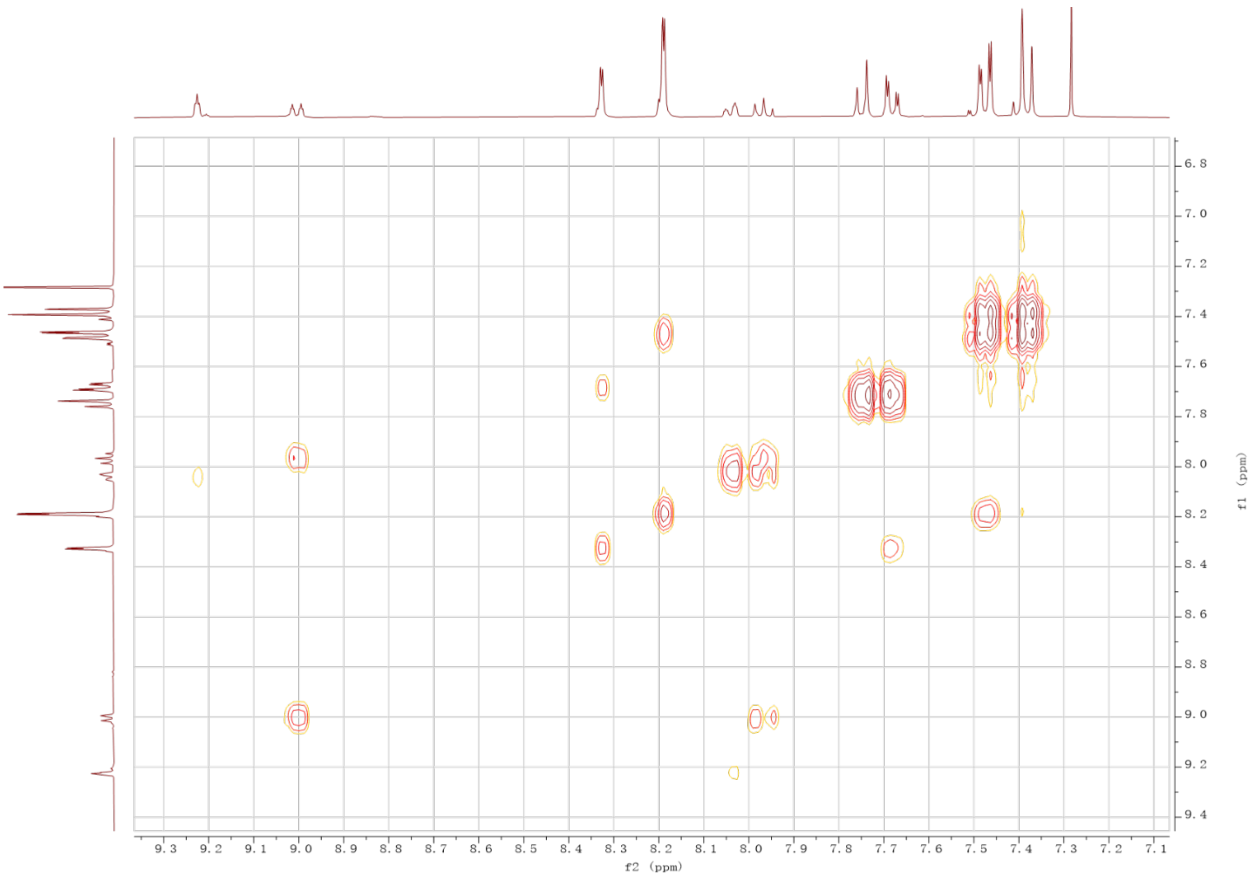

Figure S18. ^{1}H-^{1}H COSY NMR spectrum of **tBuCz3mTRZ** in $CDCl_3$.

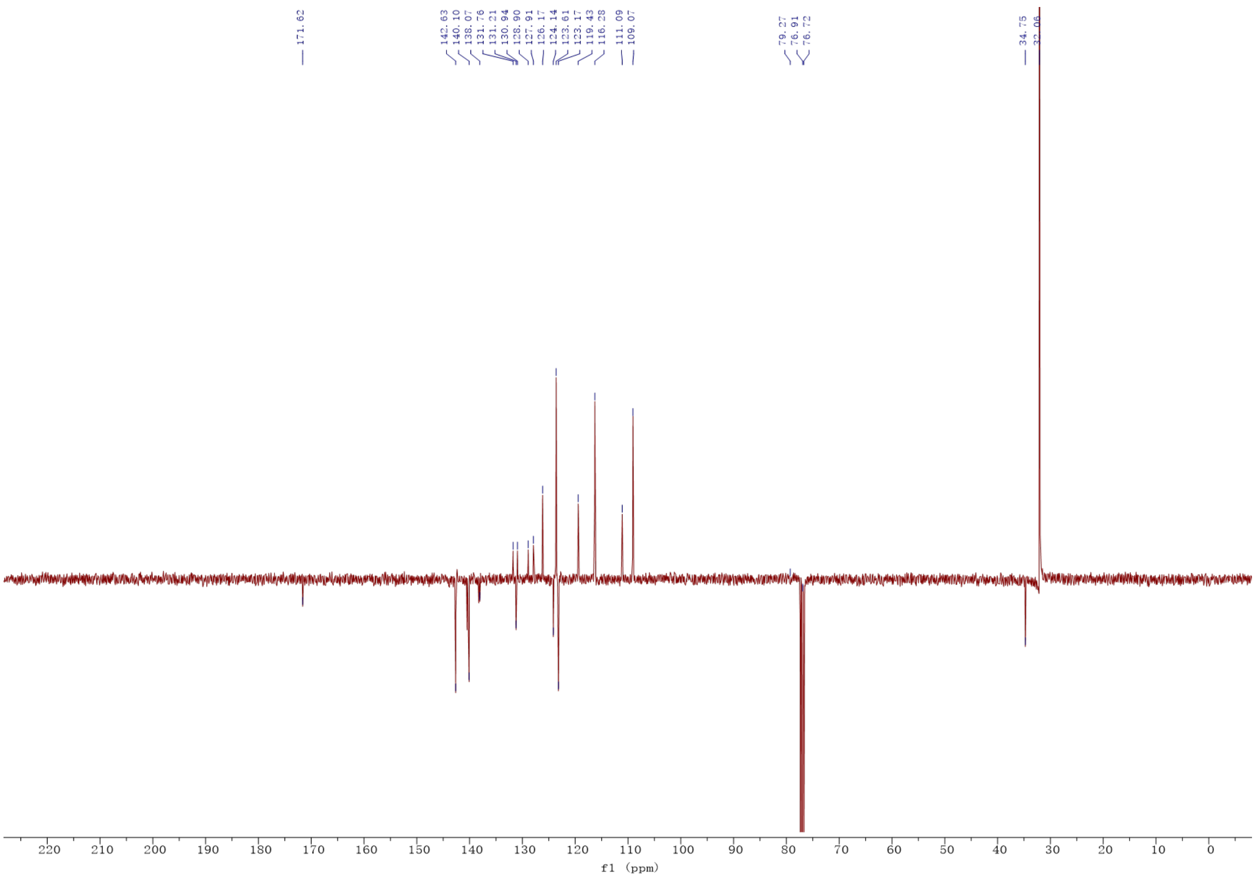

Figure S19. DEPTQ-135 NMR spectrum of **tBuCz3mTRZ** in $CDCl_3$.

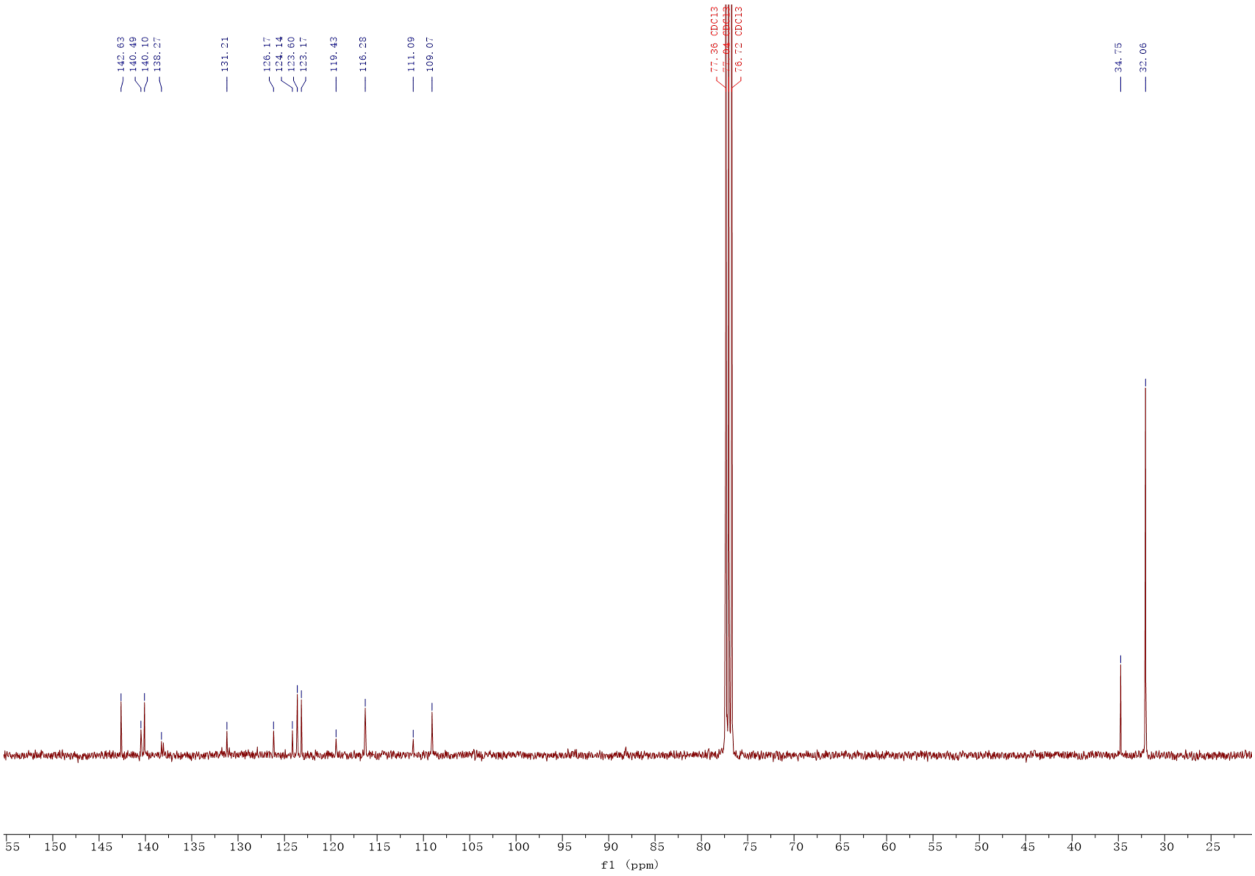

Figure S20. ^{13}C NMR spectrum of **tBuCz3mTRZ** in $CDCl_3$.

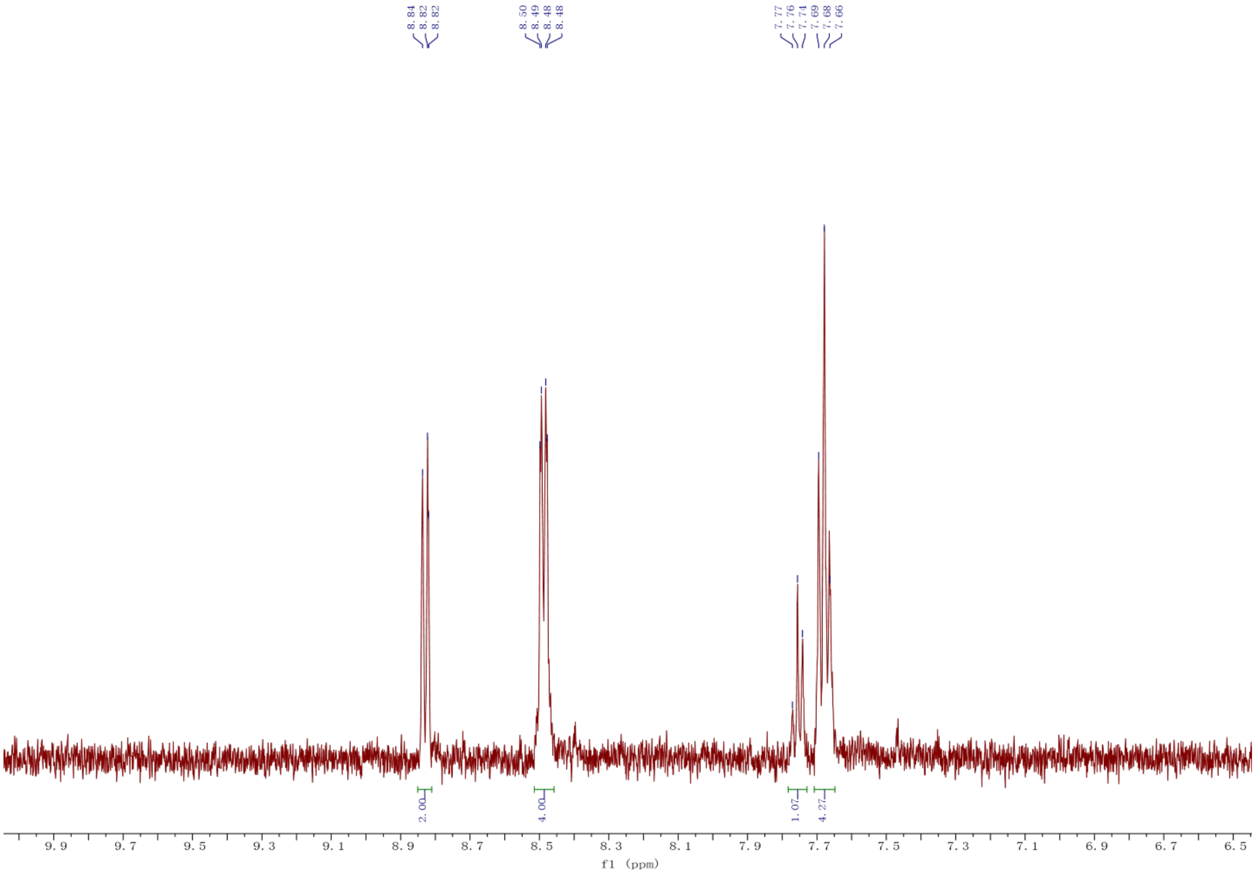

Figure S21. ^{1}H NMR spectrum of *2,4-bis(3,5-difluorophenyl)-6-phenyl-1,3,5-triazine* in DMSO.

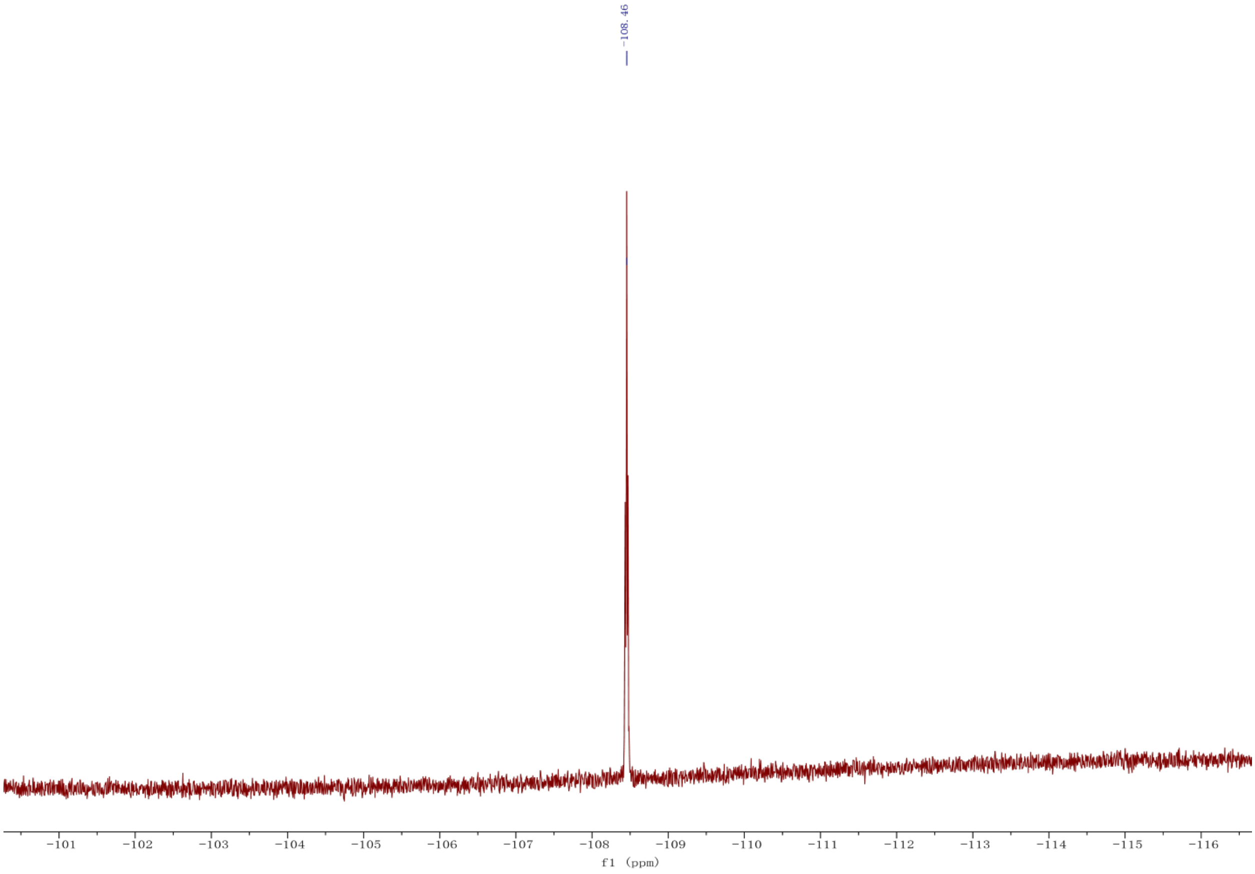

Figure S22. ^{19}F NMR spectrum of *2,4-bis(3,5-difluorophenyl)-6-phenyl-1,3,5-triazine* in DMSO.

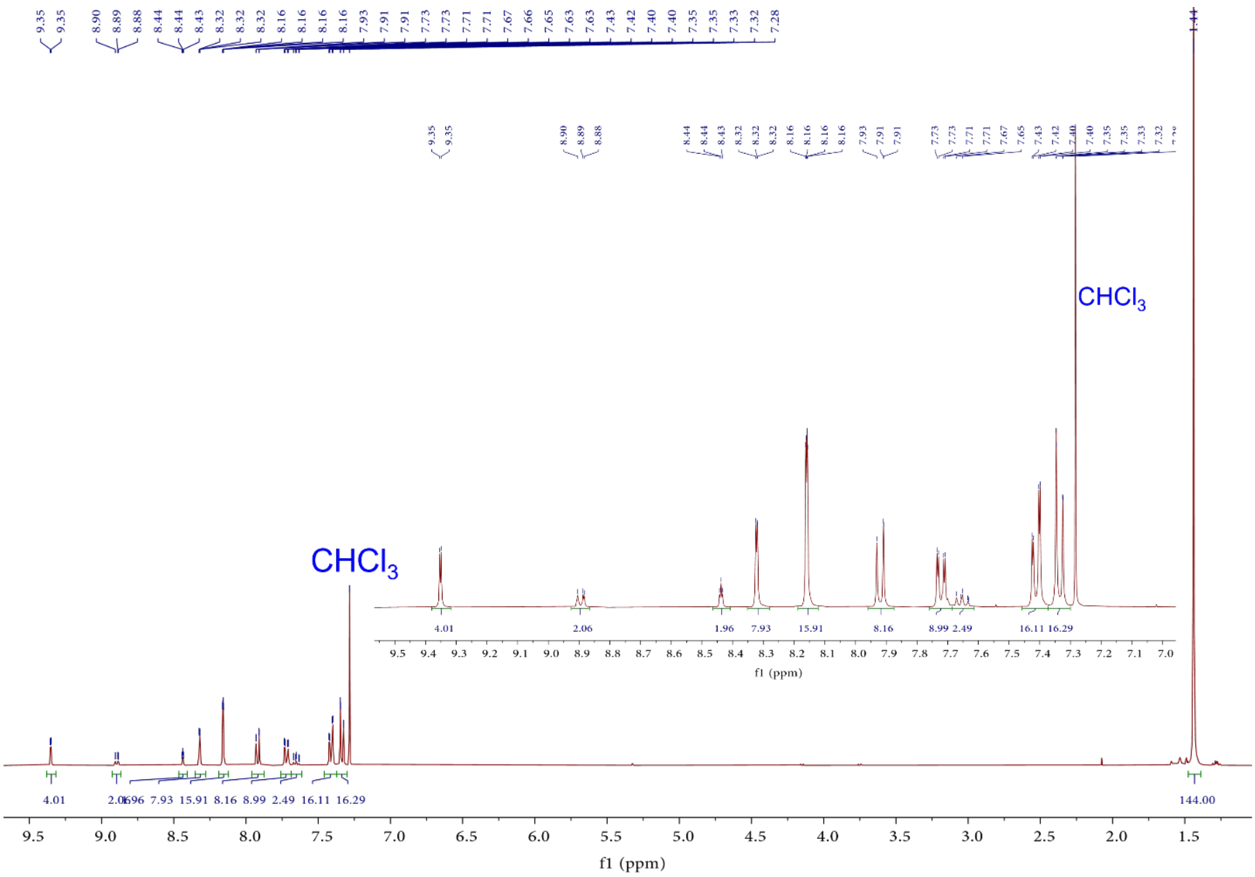

Figure S23. ¹H NMR spectrum of **tBuCz4mTRZ** in $CDCl_3$.

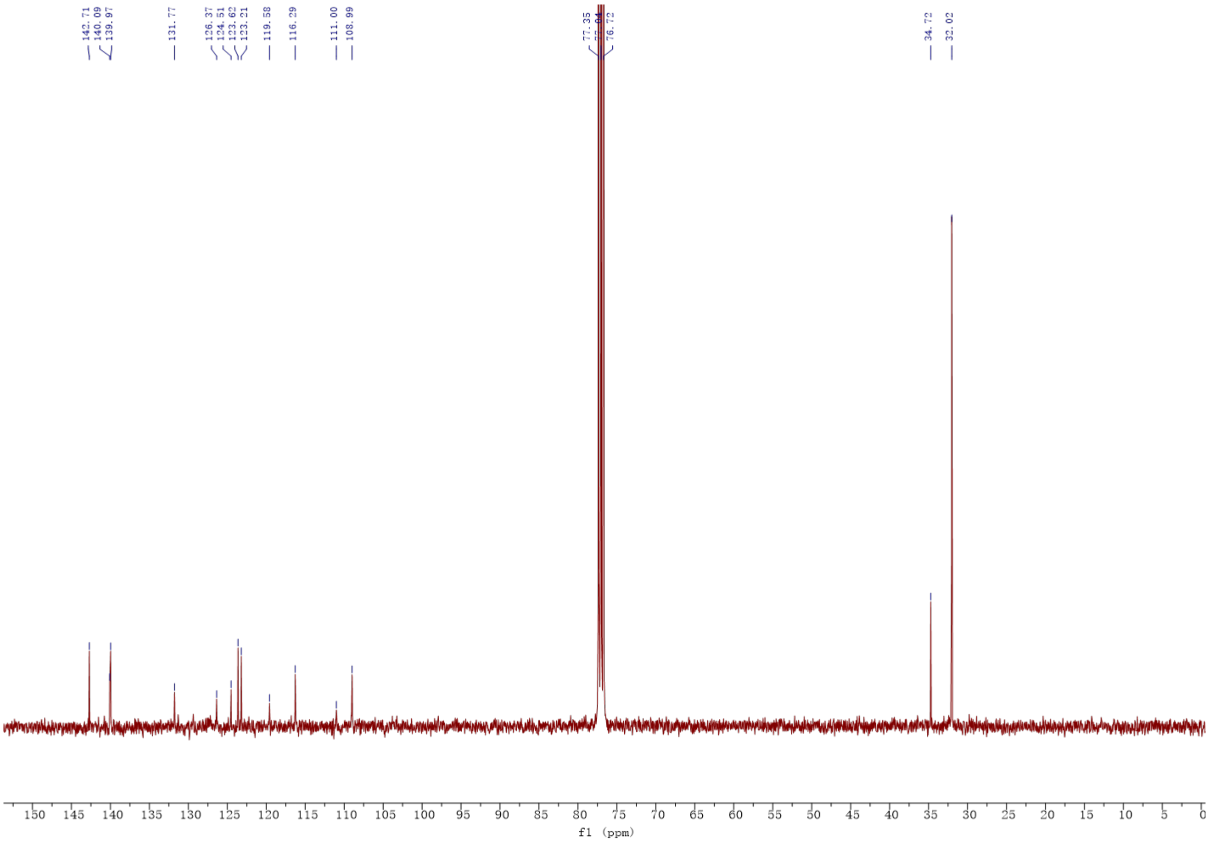

Figure S24. ^{13}C NMR spectrum of **tBuCz4mTRZ** in $CDCl_3$.

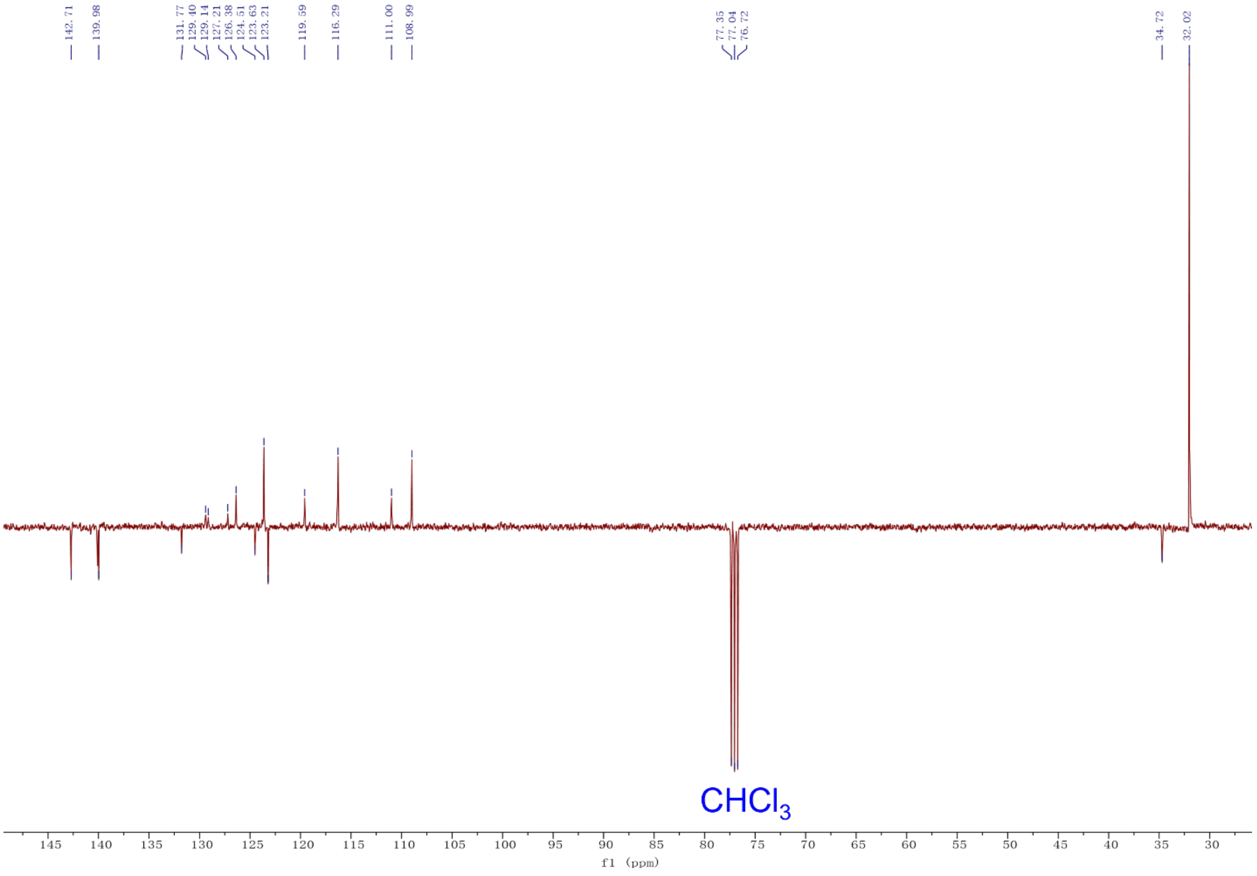

Figure S25. DEPTQ 135 NMR spectrum of **tBuCz4mTRZ** in $CDCl_3$.

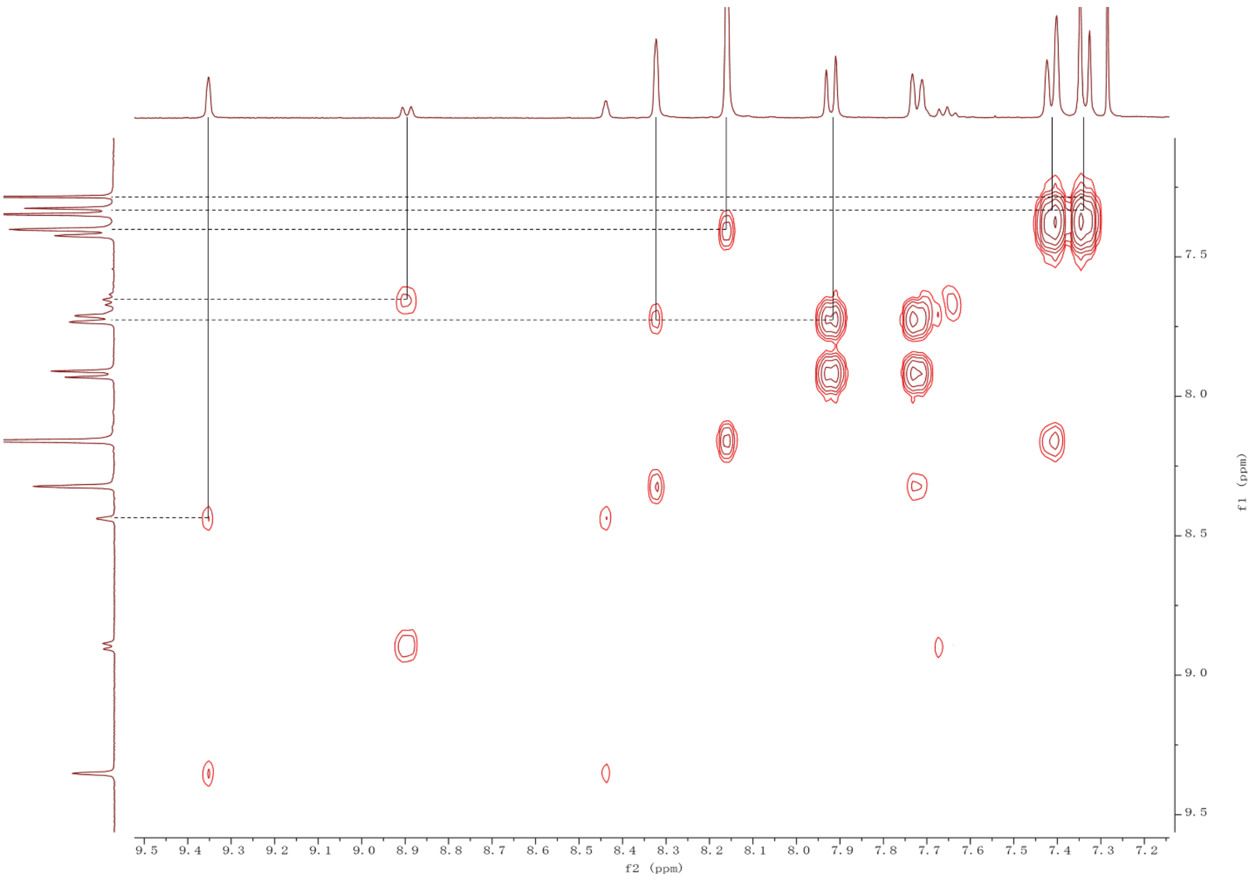

Figure S26. ^{1}H-^{1}H COSY NMR spectrum of **tBuCz4mTRZ** in $CDCl_3$.

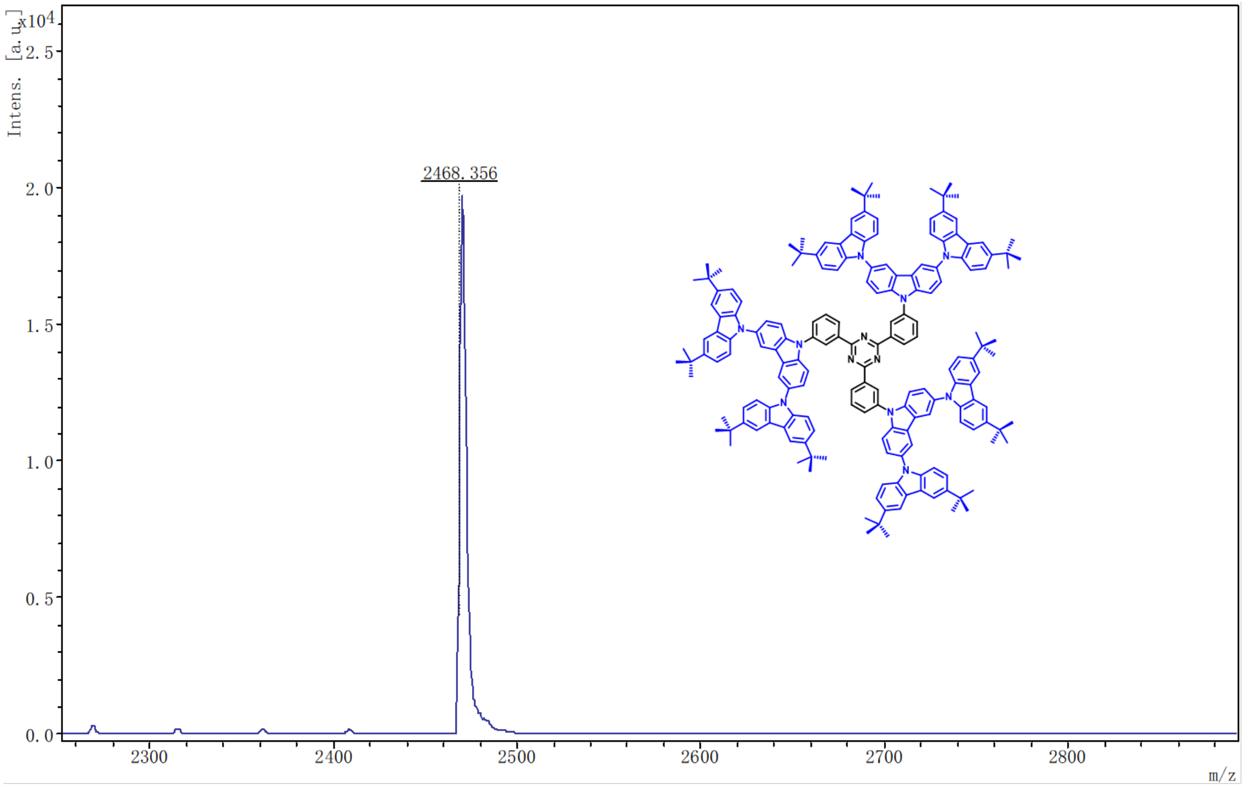

Figure S27. MALDI-TOF-MS spectrum of **tBuCz3mTRZ**.

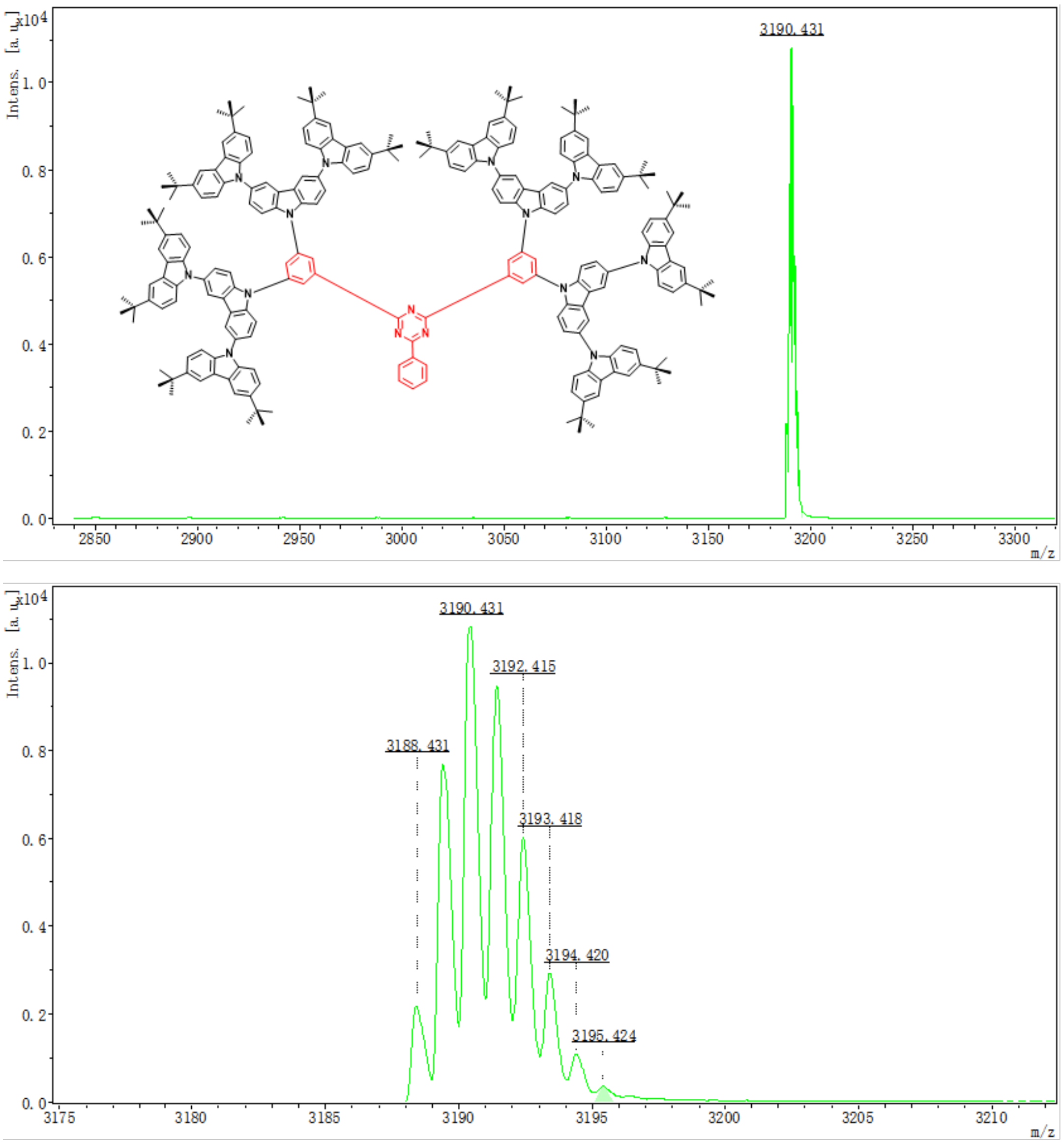

Figure S28. MALDI-TOF-MS spectrum of **tBuCz4mTRZ**.

Reference

[1] a) K. Albrecht, K. Matsuoka, D. Yokoyama, Y. Sakai, A. Nakayama, K. Fujita, K. Yamamoto, *Chem. Comm.* **2017**, 53, 2439; b) A. Sørensen, A. M. Castilla, T. K. Ronson, M. Pittelkow, J. R. Nitschke, *Angew. Chem. Int. Ed.* **2013**, 52, 11273.

[2] N. G. Connelly, W. E. Geiger, *Chem. Rev.* **1996**, 96, 877.

[3] K. Albrecht, K. Matsuoka, K. Fujita, K. Yamamoto, *Angew. Chem. Int. Ed.* **2015**, 54, 5677.

[4] K. Matsuoka, K. Albrecht, K. Yamamoto, K. Fujita, *Sci. Rep.* **2017**, 7, 1.

[5] J. Luo, S. Gong, Y. Gu, T. Chen, Y. Li, C. Zhong, G. Xie, C. Yang, *J. Mater. Chem. C* **2016**, 4, 2442.

[6] Y. Li, G. Xie, S. Gong, K. Wu, C. Yang, *Chem. Sci.* **2016**, 7, 5441.

[7] X. Ban, W. Jiang, T. Lu, X. Jing, Q. Tang, S. Huang, K. Sun, B. Huang, B. Lin, Y. Sun, *J. Mater. Chem. C* **2016**, 4, 8810.

[8] X. Ban, W. Jiang, K. Sun, B. Lin, Y. Sun, *ACS Appl. Mater. Interfaces* **2017**, 9, 7339.

[9] K. Sun, Y. Sun, W. Tian, D. Liu, Y. Feng, Y. Sun, W. Jiang, *J. Mater. Chem. C* **2018**, 6, 43.

[10] Y. Li, T. Chen, M. Huang, Y. Gu, S. Gong, G. Xie, C. Yang, *J. Mater. Chem. C* **2017**, 5, 3480.

[11] M. Godumala, S. Choi, H. J. Kim, C. Lee, S. Park, J. S. Moon, K. Si Woo, J. H. Kwon, M. J. Cho, D. H. Choi, *J. Mater. Chem. C* **2018**, 6, 1160.

[12] J. Li, X. Liao, H. Xu, L. Li, J. Zhang, H. Wang, B. Xu, *Dyes Pigm.* **2017**, 140, 79.

[13] K. Sun, D. Chu, Y. Cui, W. Tian, Y. Sun, W. Jiang, *Org. Electron.* **2017**, 48, 389.

[14] a) G. Kreiza, D. Banevičius, J. Jovaišaitė, K. Maleckaitė, D. Gudeika, D. Volyniuk, J. V. Gražulevičius, S. Juršėnas, K. Kazlauskas, *J. Mater. Chem. C* **2019**, 7, 11522; b) F. B. Dias, T. J. Penfold, A. P. Monkman, *Methods Appl. Fluoresc.* **2017**, 5, 012001.
